# Predictions of novel *Schistosoma mansoni* - human protein interactions consistent with experimental data

**DOI:** 10.1038/s41598-018-31272-1

**Published:** 2018-08-30

**Authors:** J. White Bear, Thavy Long, Danielle Skinner, James H. McKerrow

**Affiliations:** 1 Department of Bioengineering and Therapeutic Sciences, Department of Pharmaceutical Chemistry, and California Institute for Quantitative Biosciences, University of California, San Francisco, CA 94158 USA; 2 Graduate Group in Bioinformatics, University of California, San Francisco, CA 94158 USA; 3 Department of Pathology and Sandler Center for Basic Research in Parasitic Diseases, University of California at San Francisco, San Francisco, California, 94158 USA; 4 Skaggs School of Pharmacy and Pharmaceutical Sciences, University of California San Diego 9500 Gilman Dr, La Jolla, CA 92093 USA; 5 Present Address: INRA - InTheRes - UMR 1436, Equipe Transporteurs Membranaires et Résistance, 180, Chemin de Tournefeuille, Toulouse, France; 6 Present Address: MIT Lincoln Laboratory 244 Wood St, Lexington, MA USA

## Abstract

Infection by the human blood fluke, *Schistosoma mansoni* involves a variety of cross-species protein- protein interactions. The pathogen expresses a diverse arsenal of proteins that facilitate the breach of physical and biochemical barriers present in skin evasion of the immune system, and digestion of human plasma proteins including albumin and hemoglobin, allowing schistosomes to reside in the host for years. However, only a small number of specific interactions between *S. mansoni* and human proteins have been identified. We present and apply a protocol that generates testable predictions of *S. mansoni*-human protein interactions. In this study, we have preliminary predictions of novel interactions between schistosome and human proteins relevant to infection and the ability of the parasite to evade the immune system. We applied a computational whole-genome comparative approach to predict potential *S. mansoni*-human protein interactions based on similarity to known protein complexes. We first predict *S. mansoni* -human protein interactions based on similarity to known protein complexes. Putative interactions were then scored and assessed using several contextual filters, including the use of annotation automatically derived from literature using a simple natural language processing methodology. Next, *in vitro* experiments were carried out between schistosome and host proteins to validate several prospective predictions. Our method predicted 7 out of the 10 previously known cross-species interactions involved in pathogenesis between *S. mansoni* and its human host. Interestingly, two novel putative interactions involving *Schistosoma* proteins, the cercarial elastase SmCE, and the adult tegument surface protein Sm29, were also predicted and experimentally characterized. Preliminary data suggest that elafin, a host endogenous serine protease inhibitor, may be a novel substrate for SmCE. Additionally, CD59, an inhibitor of the membrane attack complex, could interact with Sm29. Furthermore, the application framework provides an integrated methodology for investigation of host-pathogen interactions and an extensive source of orthogonal data for experimental analysis. We have made the predictions available for community perusal.

## Introduction

### Etiological Agents and Effects of Schistosomiasis

*Schistosoma* are dioecious parasitic trematodes (flukes) that cause the chronic disease schistosomiasis, affecting over 230 million people worldwide and causing more than 200,000 deaths a year. They are digenetic organisms with six life cycle stages, four of which take place in the human host^1^. *Schistosoma mansoni*, one of the major etiological agents in Africa and South America of chronic schistosomiasis, releases eggs that become trapped in host tissues, triggering an unsuccessful immune response and eliciting a host granulomatous response.

The host granulomatous reaction is a primary cause of mortality associated with schistosomiasis^2,3^. Infection during childhood frequently results in growth retardation and anemia. The parasite may persist in the host for up to 40 years with a high possibility of reinfection in endemic areas^4^. Standard methods for treating schistosomiasis do not provide prophylaxis against newly acquired infections (i.e. the cercarial and schistosomula stages of the life cycle), and are locally reported as less effective even in infected adults^5–7^. Therefore, there is a need for improved and affordable treatments^7,8^.

### *S. mansoni* - Human Pathogenesis and Infection

*S. mansoni* infection involves parasite- human protein interactions over four of the six parasite life cycle stages^1^. Infection begins during the cercarial stage of the life cycle when the freshwater-dwelling larval cercariae contacts the human host. Invasion of the skin is achieved through degradation of the extracellular matrix. Cercarial elastase, one of the key enzymes identified in the process, may also help avoid the host immune response through cleavage of human C3 Complement^9^. *S. mansoni* sheds its tail to progress to the schistosomula life cycle stage, which enters the bloodstream and is carried by blood flow to the lungs and ultimately the hepatic portal system.

Using proteomic analysis, cercarial elastase was implicated in the cleavage of an extensive list of human proteins, with follow-up experiments confirming its cleavage of at least seven dermal proteins^10^. After schistosomula entry, maturation to the adult life cycle stage occurs in the inferior mesenteric blood vessels where a number of proteins aid in immune evasion and digestion of human plasma proteins^10–12^. Among the proteins expressed in the adult cycle is the adult tegument surface protein Sm29, a potential schistosomiasis vaccine candidate antigen. Sm29 interacts with unknown human immune proteins^13^. The final life cycle stage in humans is the egg phase; mated adults produce hundreds of eggs per day to facilitate transmission back to fresh water. The immune reaction to eggs leads to schistosome pathogenesis^1–4^.

#### Large-Scale Computational Prediction

Ongoing efforts to address schistosomiasis include the development of new vaccines. Knowledge of the specific protein-protein interactions between the pathogen and human host can greatly facilitate this effort. However, a comprehensive literature review revealed only ten confirmed interactions that could be predicted using our protocol, indicating the characterization effort is still in progess. These interactions were identified by experiments such as *in vitro* Edman degradation^10^, fluorescence end point assay^14^, crystallography^15^, and measurement of released radioactivity from a suspension^16^. Further types of low-throughput experiments could be based on hypothesis of specific predicted protein interactions^10^.

While many methods have been developed to predict intraspecies protein-protein interactions, few have focused specifically on interspecies interactions, where knowledge of the biological context of pathogenesis can be used to refine predictions. Previous work developed a protocol to predict interactions and applied these in the host-pathogen context^17,18^. Host-pathogen protein complexes were identified using comparative modeling based on a similarity to protein complexes with experimentally determined structures. The binding interfaces of the resulting models were assessed by a residue contact statistical potential, and filtered to retain the pairs known to be expressed in specific pathogen life cycle stages and human tissues (i.e. Biological Context Filter), thus increasing confidence in the predictions. The host-pathogen prediction protocol was benchmarked against known complexes and applied to predict interactions between human and ten different pathogenic organisms^17,18^.

#### Informing Computational Predictions

A crucial step for the construction of the Biological Context Filter is to annotate pathogen proteins by the life cycle stages in which they were expressed. This step is especially informative for *S. mansoni*, a digenetic organism, with life cycle stages and protein expression specific to both the mollusc and human hosts. While the human genome has been extensively annotated and made generally available, pathogen genome and expression annotation can be more elusive. Pathogens such as *Plasmodium falciparum* have been sequenced and extensively annotated^19^. In comparison, the *S. mansoni* genome, while much larger than that of *P. falciparum* (11,809 *S. mansoni* proteins vs 5,628 *P. falciparum* proteins), was only recently sequenced and assigned a full set of accession identifiers in GeneDB^20^. The sequencing effort is ongoing and there is limited annotation of corresponding proteins and structural information available. Thus, it is challenging even to cross-reference *S. mansoni* proteins described in various reports, particularly in those published prior to full genome sequencing^21^. Additionally, life cycle stage annotation is difficult to access or even absent in most databases.

The best source of annotation is directly from primary references in literature. Most primary reference accessions were often embedded in portable document format (pdf) files and other file formats that make extraction challenging. Furthermore, the context of an extracted accession and the life cycle stage to which it applies must be isolated from each reference and verifiable for accuracy and further study. We address these challenges by designing a simple natural language processing engine (NLP) that accomplishes data extraction, accuracy, verifiability, and correlation between disperse data sets required to construct the Biological Context Filter.

In Results below, we describe the benchmarking of our host-pathogen prediction protocol against previously identified interactions, followed by experimental characterization of putative predictions between host-pathogen proteins that indicate novel interactions.

## Results and Discussion

### Protein Interaction Prediction

**Figure 1:**
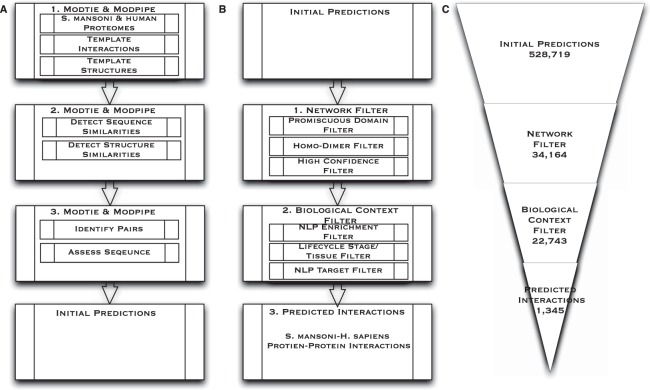
Prediction Framework. (**A**) Modtie & ModPipe protocol for detecting sequence and structure similarity: 1. The protocol begins with the set of human and *S. mansoni* proteins. 2. Sequence matching procedures are then used to identify similarities between the proteins and proteins with known structure or interactors. 3. A structure-based statistical potential assessment, or a sequence similarity score in the absence of structure, is then used to identify pairs with similarity to known complexes, assess the basis for a putative interaction, predict interacting partners and yield the Initial Predictions. (**B**) Initial Predictions: 1. Network Filter: Promiscuous Dimers, homo-dimer complexes, and high confidence interactions are then extracted from the initial set of predictions. 2. The Biological Context Filter is applied to the remaining set of predictions, weighting and ranking NLP (natural language processing) enriched predictions, isolating life cycle stage and tissues interactions between *S. mansoni* and humans, and application of the Targeted Filter. 3. Predicted Interactions are an output of the Biological Context Filter. (**C)** This Illustrates the numerical reduction in interactions obtained after each step. The framework reduces the number of potential *S. mansoni*-human protein interactions by about three orders of magnitude as shown here and in Tables 1–3.

The protocol begins with the initial set of 3,052 *S. mansoni* and 8,784 human protein sequences (Fig. 1) for which high-quality models could be created using MODTIE^18^. The initial interaction predictions (Initial Predictions) were obtained by assessing host-pathogen interactions for which comparative models could be constructed. In previous work, the fraction of pathogen proteins that aligned to a protein template of previously observed solved complex structures averaged 21%^18^. Here, only 13.9% of *S. mansoni* proteins could be modeled using such a template. Human proteome interaction template coverage remained consistent with previous work at 34%. Overall, the protocol predicted 528,719 cross-species initial potential interactions between *S. mansoni* and human proteins with similarity to solved complex structures. (Table 1).

**Table 1:** Network Filter: Interactions removed during each application of indicated filter.

Network Filter		
Filter	Number of Interactions Removed	Interactions Remaining
Unfiltered	—	528,719
Promiscuous Domains	242,677	286,042
Homo-dimer Complexes	143,065	142,977
High Confidence	108,813	34,164

Unfiltered interactions from the initial result set are shown in the first row. The number of interactions removed and remaining from each filtering step are shown in each category column including Promiscuous Pairs, Homo-dimer Complexes, and High Confidence interactions. The remaining 34,164 interactions are used as an input into the next step, the Biological Context Filter.

We applied three network-level filters to prune the initial predictions (Fig. 1). The first Network Filter removed interactions where the templates are used for more than 1% of the total predictions. These include templates based on promiscuous domains and sequences that can present as promiscuous due to similarity or other reasons. Promiscuous domains, while present in many interacting complexes lack specificity and are overrepresented in the predicted data set, making them less desirable as vaccine candidate antigens. For example, a domain in the crystal structure of HIV Capsid Protein (p24) bound to FAB13B5, Protein Data Bank (PDB 1E6J), is a frequently used template in potential interactions. Fab (fragment antigen binding) regions, like FAB13B5, are immunoglobin proteins which form the paratope. They are highly variable in sequence and composed of less than 22 amino acids. Many templates will score above the alignment threshold for this portion of the paratope and the short protein sequence acting as the epitope in the binding site. Shorter sequence lengths, high variability, and conservation across species increases the likelihood of similarity and results in a disproportionate number of potential interactions. Many variations will either not be applicable to *S. mansoni* and human interactions or conserved across species for binding similar epitopes and would be overrepresented. They are not indicative of an interaction between our target complexes because of the lack of specificity. Templates, similar to p24 bound to FAB13B5, were too generalized to draw conclusions with any degree of confidence and removed as promiscuous domains in the first Network Filter^22,23^.

In the second Network Filter, predictions based on homodimer complexes were removed. This step removes instances of highly conserved interacting dimers from similar complexes in both *S. mansoni* and humans occur due to speciation events^24,25^. An example of such is the FGFR2 tyrosine kinase domain (PDB 1E6J). FGFR2 has high similarity to tyrosine kinases in *S. mansoni*, but were generally conserved in eukaryotes and thus comprise a bias in the homodimerization interaction of the catalytic subunits^26^. In the final Network Filter step, interactions with less than a 97% confidence interval were removed to further narrow the focus of potential interactions and obtain a higher confidence level (Materials & Methods). This filtering results in the remaining 34,164 interactions (Table 1).

Next, the Biological Context Filter isolates potential interactions according to various life cycle stages of *S. mansoni* and likelihood of *in vivo* occurrence in human tissues. In the first step of the Biological Context Filter, interactions passing the Network Filter were enriched with data from a simple natural language processing (NLP) algorithm that automatically identified *S. mansoni* proteins expressed in different pathogen life cycle stages from literature and databases using listed nomenclature and functional information (Materials & Methods) (Table 2). However, there is limited life cycle stage expression information using database annotation.

**Table 2:** Biological Context Filter: Interactions removed during each application of indicated filter.

Biological Context Filter Interactions		
Filter	Interactions Removed	Interactions Remaining
Network	—	34,164
NLP & Enrichment	10,929	23,235
Life cycle/Tissue	492	22,743
Targeted	21,398	1,345

Unfiltered interactions from the Network Filter result set are shown in the first row. The number of interactions found from the total resulting data set are shown in each category column.

The NLP algorithm was designed to address this limitation and accomplished the following: (1) characterization of 12,720 *S. mansoni* genes automatically from primary reference; (2) recording of contextual, life cycle stage, and citation information into a customized database; and (3) programmatic correlation of this data with existing database annotations. This resulted in annotation of 96.6% of *S. mansoni* sequences, greatly exceeding existing annotation from any single database, which topped out at 62%. NLP annotation further extended this coverage with life cycle stage and characterization information not readily available in database annotation.

Next, the Life Cycle/Tissue Filter refines interactions for likelihood of *in vivo* interaction based on biological context derived from NLP of the component proteins in each interacting complex and their expression in each of the four life-cycle stages of *S. mansoni* in different human tissues (Materials & Methods). A list of pathogen life cycle stage and human tissue pairs was generated (Table 3).

**Table 3:** Protein interactions involved in *S. mansoni* life cycle stages that are directly involved in human pathogenesis from the resulting targeted predictions are shown with targeted life cycle stage, the number of predicted interactions for the corresponding life cycle stage and associated human tissues involved in the interaction.

Biological Context Filter Interactions and *S. mansoni* Life Cycle Stage		
Life cycle stage	Correlated Interactions	Life cycle stage-Tissue
Cercariae	460 (460/1345)	1 (skin)
Schistosomula	442 (442/1345)	15
Adult	329 (329/1345)	13
Egg	114 (114/1345)	11

Human tissue expression data were obtained from the GNF Tissue Atlas^53^ and GO^56^ functional annotation unless noted otherwise.

The third Biological Context Filter applies a targeted post-process analysis of potential interactions. In this step, NLP parameters were used to rank the prediction based on number of occurrences of the interacting proteins and the assigned weight of the literature where the observations occurred (Materials & Methods).

In previous work predicting host-pathogen protein interactions, filters resulted in a wide range of reductions for different pathogen genomes due to varying levels of biological annotation available for each genome. The majority of the biological annotations in Davis *et al*.^18^ were not relevant in a pathogenic context and therefore did not pass the filtering, while pathogen proteins had limited life cycle stage annotation resulting in multiple host-pathogen data sets with no interactions^18^.

In the current framework, 22,743 (Table 2) interactions passed both biological and network-level filters, which was 51.5% more than the average of the ten pathogens in the previous work despite a below average model coverage. This increase is largely due to the NLP annotation, which produced a large number of pathogen proteins with a defined life cycle stage. Overall, the Biological Context Filter resulted in 1,345 annotated interactions likely to occur *in vivo* in *S. mansoni* life cycle stage and human tissue interaction sites (Table 3).

### Assessment I: Known Interactions

To assess the predictions, we first compared the predicted set with the set of known *S. mansoni*-human protein interactions. There were 10 confirmed interactions between *S. mansoni* and human proteins. Among the 10, there is only one structure available in PDB (Table 4). The host-pathogen application framework recovered 7 of the 10 known interactions. The majority (7/10) of experimentally characterized *S. mansoni*-human protein interactions involve the serine peptidase cercarial elastase). Several experiments have characterized the cleavage by cercarial elastase of extracellular membrane and complement proteins^10,27–30^.

**Table 4:** Confirmed protein-protein interactions indicated in pathogenesis between *S. mansoni* and human proteins.

Comparison of known and predicted *S. mansoni* protein interactions				
*S. mansoni* Protein	Human Protein	Predicted	Reference	PDB
Smp-001500 EIF4E	EIF4E-binding protein 1	No	^15^	3HXG
SmCE Cercarial elastase	Collagen (I, IV, VIII)	Yes	^16, 27, 28^	2CHA
SmCE Cercarial elastase	IgE	No	^14^	—
SmCE Cercarial elastase	Complement C3 (C3b)	Yes	^10, 60^	1EQ9
SmCE Cercarial elastase	Laminin	Yes	^16, 27, 28^	2CHA
SmCE Cercarial elastase	Fibronectin	Yes	^16, 27, 28^	2CHA
SmCE Cercarial elastase	Keratin	Yes	^61^	—
SmCE Cercarial elastase	Elastin	Yes	^62, 63^	1FON
SmCB2 Cathepsin B	Collagen (I) (nidogen)	Yes	^10^	1STF
SmCB2 Cathepsin B	Complement C3	No	^10^	—

The application framework predicted 7 of the 10 known interactions indicated in pathogenesis that should have been detected by our framework. Interactions shown here are not representative of all cross-species interactions between *S. mansoni* and human proteins, but represent a selection of interactions that should have been detected given our methodology. For instance, interactions necessarily removed during filtering would not be listed in this table. Proteins are listed by common name and associated accession when available. PDB column indicates the template obtained from PDB (http://www.rcsb.org/) structure used to predict the interaction. A dash indicates no PDB structure was available. Human tissue expression data were obtained from the GNF Tissue Atlas^53^ and GO^56^ functional annotation unless noted otherwise.

**Figure 2:**
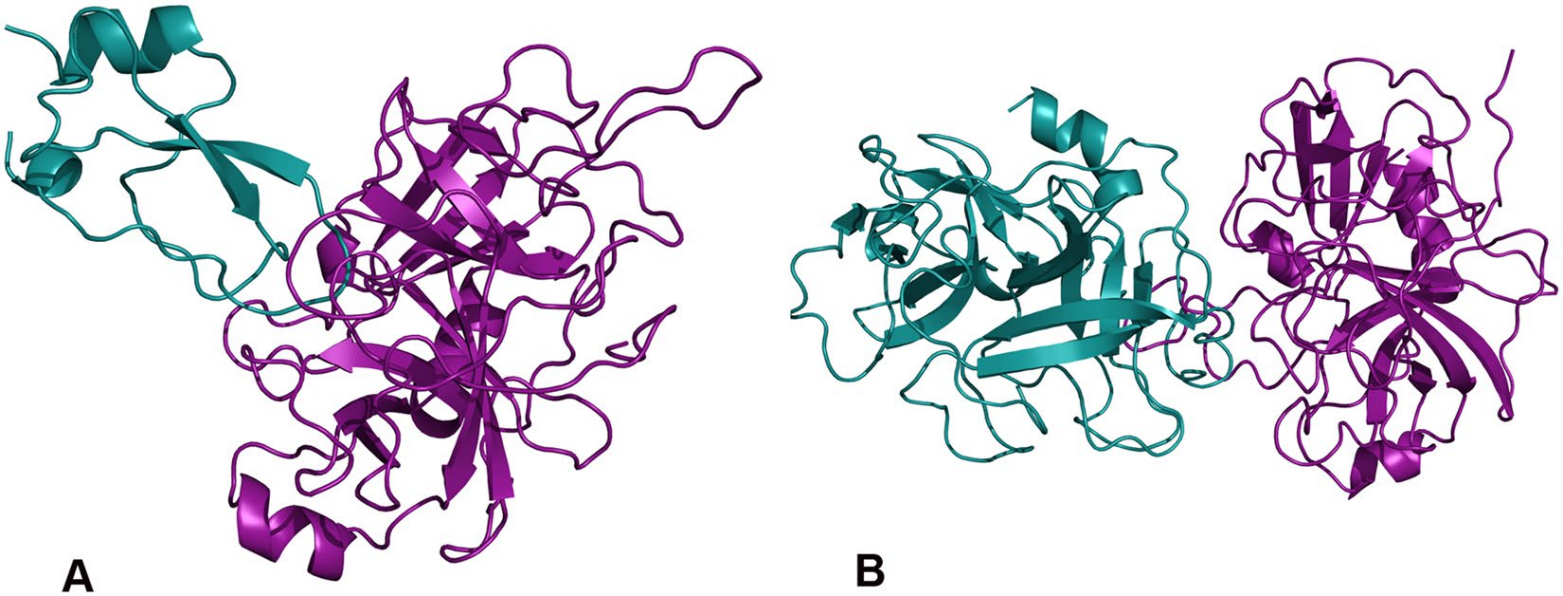
Retrospective Predictions. Examples of validated interactions. (**A**) Cercarial elastase (purple) and human collagen (blue) based on the template structure of tick tryptase inhibitor in complex with bovine trypsin (PDB 2UUY) (**B**) Cercarial elastase (purple) and human Complement C3 (precursor C3b) (blue) based on the template structure (PDB 1EQ9) of fire ant chymotrypsin complexed with PMSF, an inhibitor. Figures were generated by PyMOL (http://www.pymol.org).

Our method recapitulated several of these interactions. For example, a retrospective prediction was made between the enzyme and human collagen based on the template structure of tick tryptase inhibitor in complex with bovine trypsin (PDB 2UUY) (Fig. 2). Previous studies indicate that the enzyme has a role in suppressing host immune response (Table 4)^10^; its similarity to tryptase, which has been used as an indicator of mast cell activations and an important mechanism of host defense against pathogens^31^, is consistent with this suggested role of cercarial elastase in pathogenesis.

In addition to cercarial elastase’s cleavage of extracellular proteins, several important protein-protein interactions involved in *S. mansoni* immune evasion have been characterized, including its cleavage of Complement C3 (Table 4). Our method retrospectively predicted this interaction based on the structure of fire ant chymotrypsin in complex with the PMSF inhibitor (PDB 1EQ9) (Fig. 2). Fire ant chymotrypsin, which is similar to elastases in many species, degrades proteins for digestion and is a known target for blocking growth from the ant larval stage to adult in ant-infested areas^32^.

### Assessment II: Known Vaccine Candidate Antigens

Next we assessed predictions against experimentally characterized vaccine candidate antigens where the mechanism, specificity, and interacting human proteins were still undetermined. Currently, there are 9 *S. mansoni* proteins considered as vaccine candidate antigens and 5 protein groups viewed as vaccine candidate antigens. We predicted interactions with 5 of the current vaccine candidate antigens and all of the potential vaccine candidate antigens (Table 5).

**Table 5:** Prospective protein-protein interactions between *S. mansoni* and human proteins.

Potential *S. mansoni* Protein Interactions				
*S. mansoni* Protein	Human Protein	Predicted	Reference	PDB
Sm-TSP-1 Tetraspanin	IgG1/IgG3 Immune Response	No	^64^	—
Sm-TSP-2 Tetraspanin	IgG1/IgG3 Immune Response	No	^64^	—
Sm 29 Transmembrane	67782326 (GI) TGF-beta receptor	Yes	^13, 64^	2I9B, 1YWH
Sm 29 Transmembrane	67782324 (GI) TGF, beta receptor II	Yes	^13, 64^	2I9B, 1YWH
Sm 29 Transmembrane	42716302 (GI) CD59 glycoprotein precursor, MAC	Yes	^13, 64^	2I9B, 1YWH
Sm 29 Transmembrane	9966907 (GI) SLURP-1	Yes	^13, 64^	2I9B, 1YWH
Sm 29 Transmembrane	4505865 (GI) PLAU	Yes	^13, 64^	2I9B, 1YWH
Sm 29 Transmembrane	53829381 (GI) PLAU	Yes	^13, 64^	2I9B, 1YWH
Sm 29 Transmembrane	53829379 (GI) PLAU	Yes	^13, 64^	2I9B, 1YWH
Sm 29 Transmembrane	4504033 (GI) GPI anchored molecule-like	Yes	^13, 64^	2I9B, 1YWH
Sm 23 Tetraspanin	IgG3, MAP-3 Immune Response	No	^13, 64– 66^	—
Sm 14 FABP	IgG1, IgG3 Immune Response	No	^13, 29, 59, 64, 66– 69^	—
Sm 97 Paramyosin	IgG, IgE Immune Response	Yes	^66, 68, 70^	—
Sm 28 GST	IL-5, IgG2, MAP-4 Immune Response	No	^13, 66, 68^	—
SOD SOD [Cu-Zn], Cytosolic	4507149 (GI) SOD1 [Cu-Zn]	Yes	^13, 59, 66, 71^	2AF2, 1JK9
SOD SOD [Cu-Zn], Cytosolic	118582275 (GI) SOD3 Extracellular	Yes	^13, 59, 66, 71^	2AF2
SOD SOD [Cu-Zn], Cytosolic	4826665 (GI) Copper chaperone for SOD	Yes	^13, 59, 66, 71^	2AF2, 1JK9
Sm-p80 Katanin p80 WD40	C3 Complement Immune Response	No	^64, 72^	—
Cercarial Elastase	4505787 (GI) Elafin *Supplemental Table 1	Yes	^35^	1FLE
Venom Allergen Proteins (VAL)	*Supplemental Table 2	Yes	^29, 64, 73^	*
Calpain	*Supplemental Table 3	Yes	^13, 30, 66, 72, 73^	*
Cystatin	*Supplemental Table 4	Yes	^59, 66^	*
Tetraspanin	*Supplemental Table 5	Yes	^13, 64, 73, 74^	*
Immune evasion	Immunoglobin Proteins *Supplemental Table 6	Yes	^5, 13, 14, 36, 59, 75^	*

Prospective interactions are hypothesized, but have little or no experimental evidence, and are currently under investigation as candidate antigens or potential vaccine candidate antigens. The application framework predicted interactions between several proteins and suggested *S. mansoni* and human interactions with further interactions listed in Supplemental Tables (1–6). Proteins are listed by common name and associated accession when available. PDB column indicates the template obtained from PDB (http://www.rcsb.org/) structure used to predict the interaction. A dash indicates no PDB structure was available. *Indicates further results listed in the Supplemental Tables. Human tissue expression data were obtained from the GNF Tissue Atlas^53^ and GO^56^ functional annotation unless noted otherwise.

We now describe two specific examples of predicted interactions involving *S. mansoni* protein vaccine candidate antigens that, after experimental follow-up, are consistent with the presented hypotheses. As noted, cercarial elastase is known to cleave several human proteins (Table 4), and it is considered a vaccine candidate antigen due to its abundance in *S. mansoni* cercarial secretions^33^. Functionally, it has been indicated as the primary means of pathogen entry across the human dermal barriers, the first stage of pathogenesis^27^.

**Figure 3:**
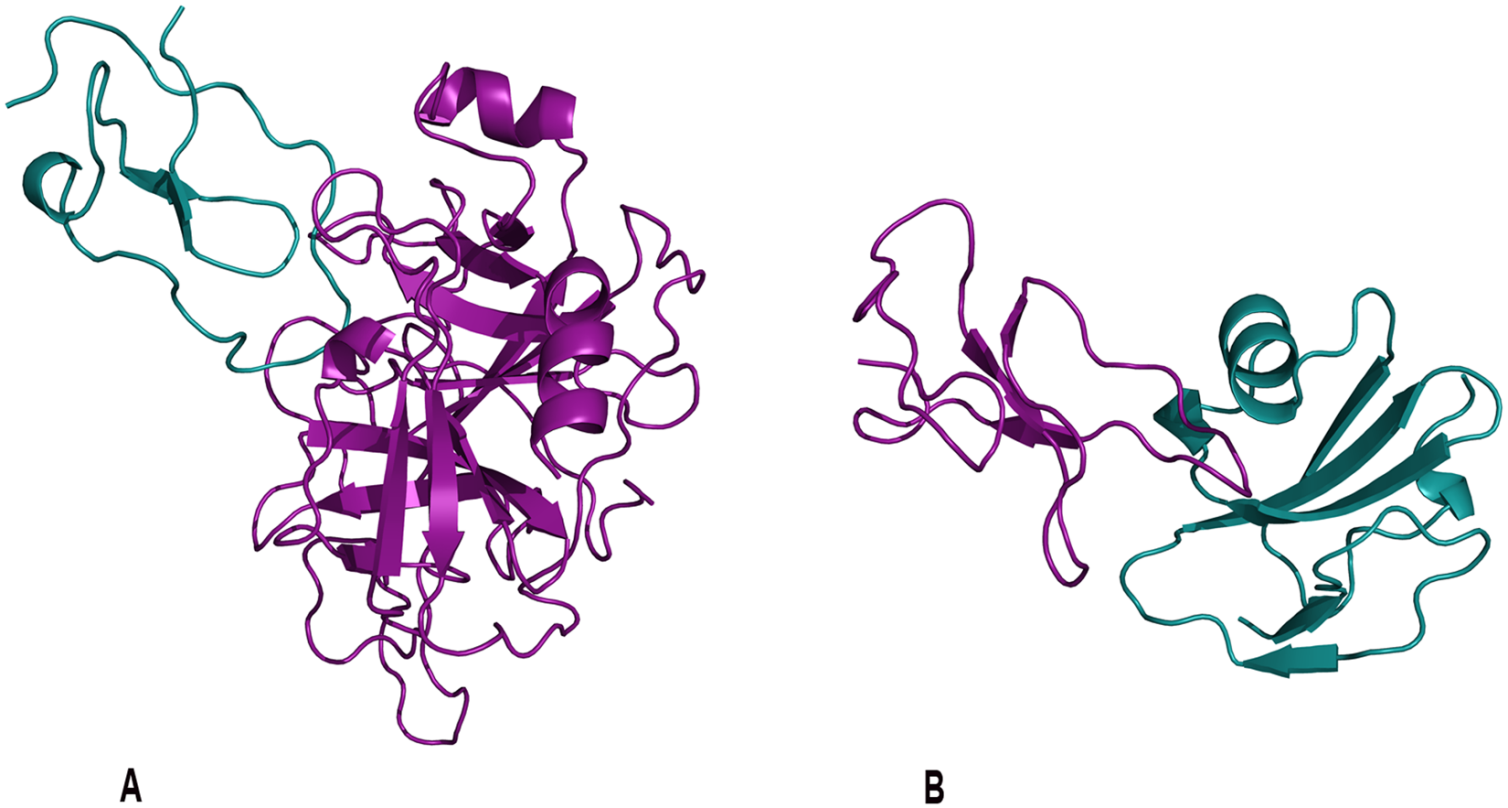
Prospective Predictions. Examples of predicted interactions. (**A)** Cercarial elastase (purple) was predicted to interact with the human elastase specific inhibitor elafin (blue). This prediction is based on the template crystal structure of elafin complexed with porcine pancreatic elastase (PDB 1FLE). (**B**) Sm29 (purple) predicted to interact with human CD59 (blue) protein involved in the membrane attack complex corroborates hypothesis of *S. mansoni*’s ability to disable immune response. This prediction was based upon the template of ATF-urokinase and its receptor (PDB 2I9B). Sm29, an uncharacterized transmembrane protein, is a *S. mansoni* surface protein in both the schistosomula and adult life cycle stages that has been indicated in several immune response interactions making it an important vaccine candidate antigen. Figures were generated by PyMOL (http://www.pymol.org).

Novel interactions between cercarial elastase, its isoforms and other human proteins including calpains, cystatins, tetraspanins, immune and complement proteins were predicted (Table 5). The first prospective interaction selected for experimental follow up was cercarial elastase and the elastase specific inhibitor elafin. This prediction was based on the template crystal structure of elafin complexed with porcine pancreatic elastase (PDB 1FLE) (Fig. 3). Elafin plays a wound-healing role in the dermal immune response in humans and is an antimicrobial against other pathogens such as *Pseudomonas aeruginosa* and *Staphylococcus aureus*^34^. Elafin has been demonstrated to bind with high affinity to both human leukocyte elastase and porcine pancreatic elastase^35^.

The next prospective interaction selected for experimental follow up involved the *Schistosoma* protein Sm29 and the human CD59 protein. Sm29, another vaccine candidate antigen indicated in pathogenic immune evasion, was involved in several predictions. The prospective interaction with the human CD59 protein, involved in the complement membrane attack complex (MAC), would aid the ability of *S. mansoni* to disable immune response. This prediction was based upon the template of ATF-urokinase and its receptor (PDB 2I9B) (Fig. 3), which is involved in multiple patho-physiological processes. Sm29, an uncharacterized transmembrane protein, is a *S. mansoni* surface protein in both the schistosomula and adult life cycle stages that has been indicated in several immune response interactions, making it an important vaccine candidate antigen^13,35^.

CD59, also known as protectin, regulates complement, inhibits the membrane attack complex (MAC), prevents lysis and is exploited as an established immune evasion tactic used by viruses^36,37^. Murine experiments indicate immunization with recombinant Sm29 reduces *S. mansoni* parasite burdens and offers protective immunity; however, the exact mechanism has not been characterized. Further experimental characterization of the predicted interaction between Sm29 and CD59 could provide greater insight on how *S. mansoni* inhibits the MAC and additional strategies for preventing this inhibition. Additional interactions involving vaccine candidate antigens and key targets are referenced in the supplement.

### Assessment III: Experimental Characterization

**Figure 4:**
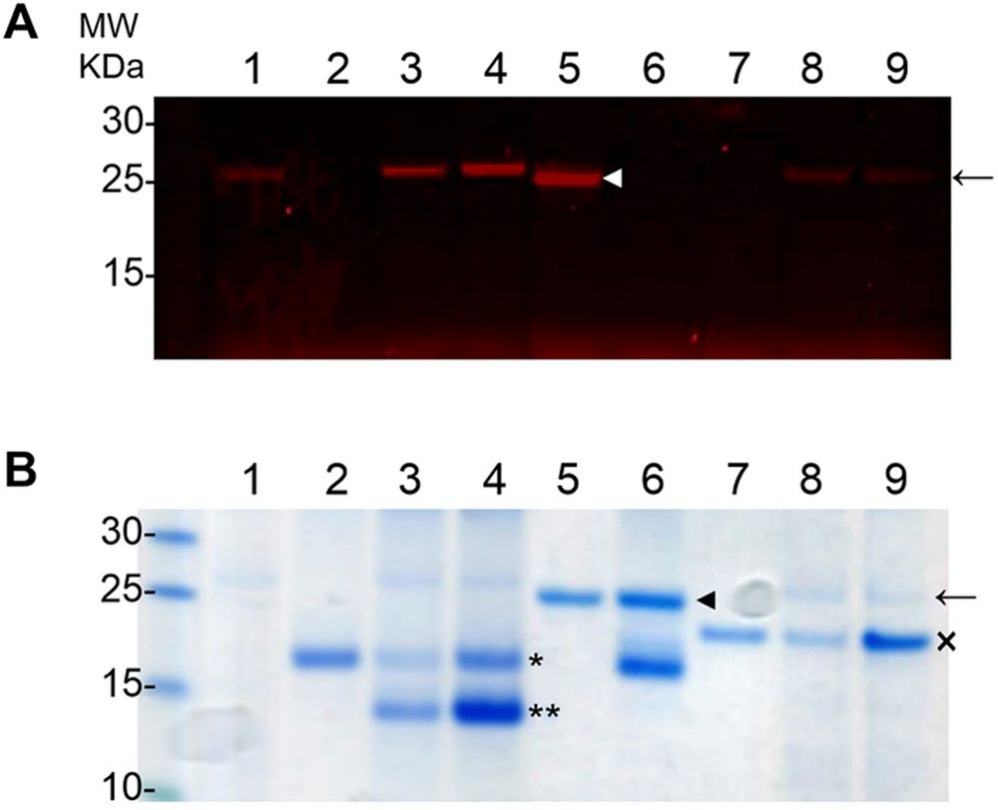
Elafin is not an inhibitor of cercarial elastase (SmCE) but is a novel substrate of SmCE (**A**) Serine protease activity assays were performed with fluorophosphanate (FP) rhodamine probe in 100 mM Tris, pH8 with (1) SmCE (arrow), (2) 54 µM elafin, (3) SmCE and 54 µM elafin, (4) SmCE and 177 µM elafin, (5) pancreatic elastase (white arrowhead), (6) pancreatic elastase and 54 µM elafin, (7) 54 µM ecotin, (8) SmCE and 54 µM ecotin, (9) SmCE and 177 µM ecotin. While elafin inhibited the activity of PE (6), it had no effects on the activity of SmCE (3,4). As control of SmCE activity assay, we showed that ecotin inhibited the activity of SmCE (8,9). (**B)** The same samples were loaded in a SDS-PAGE and stained in Coomassie blue. The incubation of SmCE (arrow) and elafin (asterisk) released an additional band (double asterisks) (3, 4), absent in the mix PE and elafin (6). This additional band increased in intensity when 177 µM were used compare to 54 µM suggesting a cleavage product of elafin by SmCE. As control, we showed that ecotin (cross) was not cleaved by SmCE (8,9).

To validate our prospective predictions (Fig. 3), we carried out *in vitro* experiments between selected schistosome vaccine candidate antigens and human proteins. First, to demonstrate any interaction between the schistosome cercarial elastase (SmCE) and elafin, we tested whether elafin was an inhibitor of SmCE as described for the pancreatic elastase (PE) by performing an *in vitro* serine protease activity of SmCE using a rhodamine-tagged fluorophosphonate rhodamine (FP-rhodamine) probe. As shown in Fig. 4A, FP-rhodamine bound to the active site of active serine proteases such as SmCE and PE alone, visualized by a fluorescent band. While elafin (54) blocked the binding of FP-rhodamine to the PE active site, there were no effects on SmCE as shown by the presence of a fluorescent band with 54 and 177 µM of elafin. Additionally, we showed that ecotin, a known serine protease inhibitor, inhibits the binding of FP-rhodamine to SmCE at 54 and 177 µM resulting in a decrease of fluorescence density^38^. These preliminary experiments suggest that elafin is not an inhibitor of SmCE, whereas elafin is a known inhibitor of PE^35^. However, follow up experiments with Sodium Dodecyl Sulfate PolyAcrylamide Gel Electrophoresis (SDS-PAGE) analysis of the SmCE activity assay indicate that SmCE interacts with elafin (Fig. 4B). Indeed, the incubation of cercarial elastase with elafin resulted in the appearance of an additional band of lower molecular weight which is absent from the pancreatic elastase assay with elafin and the controls. The density of the additional band increases with higher concentrations of elafin, from 54 μM to 177 μM confirming that this band corresponds to a fragment of elafin that is cleaved by cercarial elastase. This additional observation demonstrates that elafin is a novel substrate of SmCE emphasizing a novel interaction between SmCE and elafin that validate our prospective interaction prediction. Further experiments of the fragment released following the incubation of SmCE with elafin are needed to fully characterize this interaction. Importantly, this prospective interaction between SmCE and elafin may introduce alternative perspectives using SmCE as a vaccine candidate antigen.

**Figure 5:**
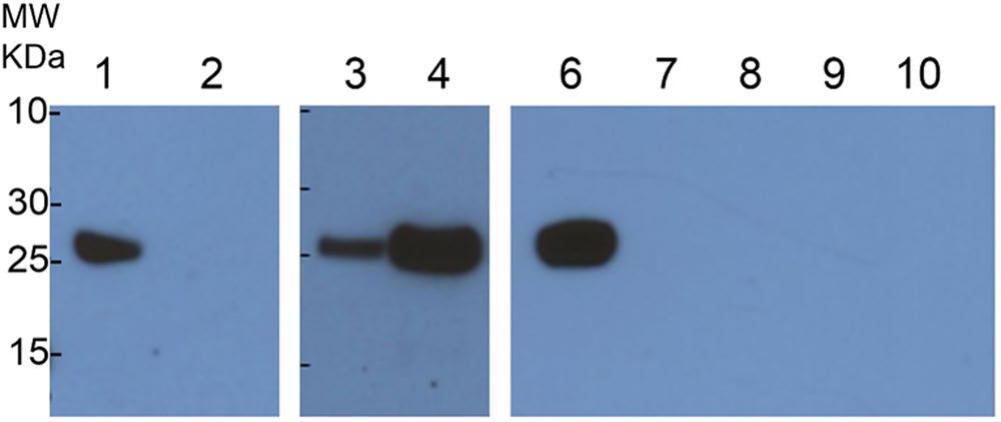
CD59 is found in freshly perfused adult worm lysates. (1) Adults worms obtained from hamster perfusion exhibited the presence of CD59 whereas (2) schistosomula obtained by mechanical method from cercariae did not show any CD59. CD59 were found in male and female adult worms and only in fresh adult worms (6). CD59 disappeared from adult worms in culture during 1 (7), 2 (8), 3 (9) and 7 (10) days.

**Figure 6:**
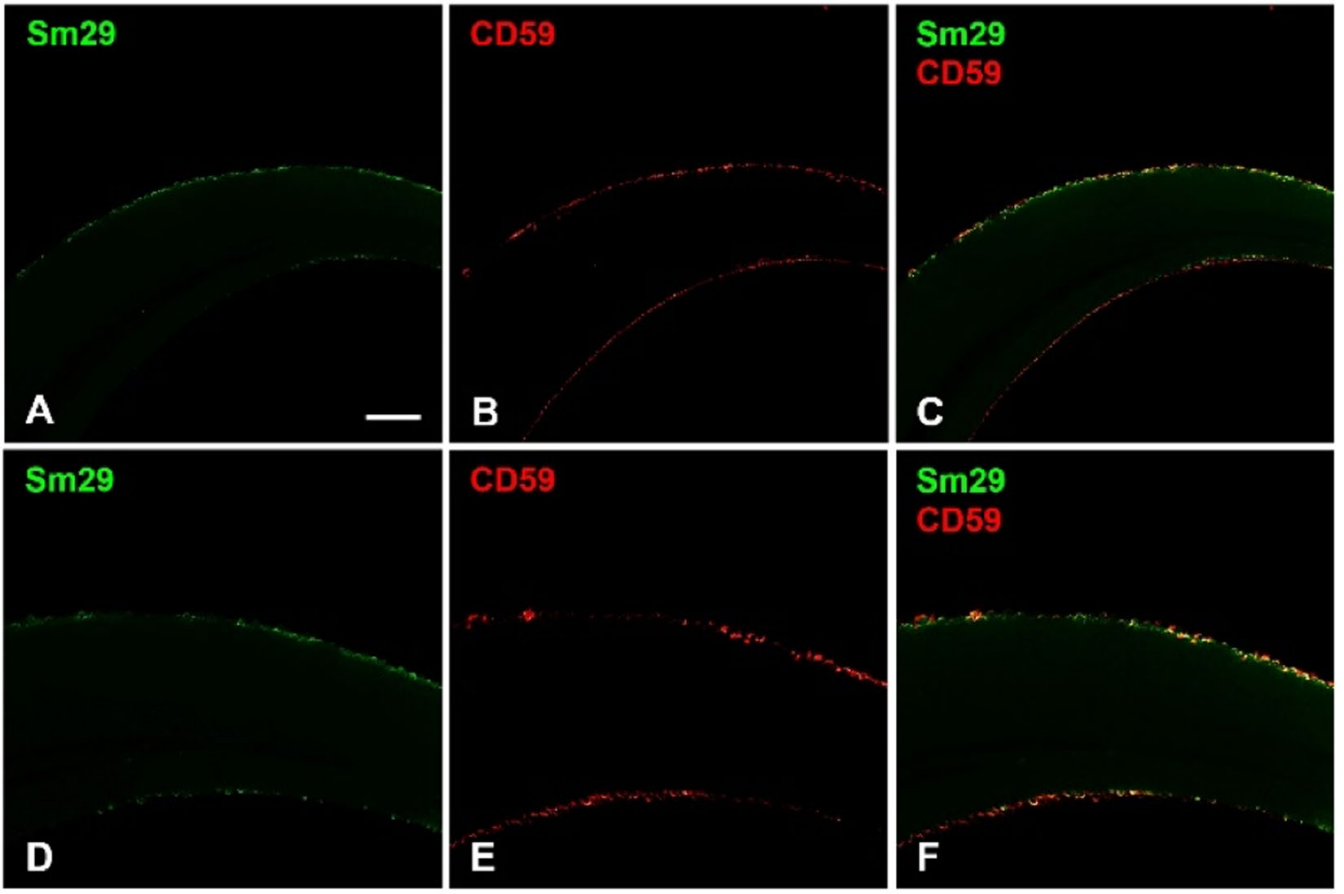
Confocal images localizing Sm29 and CD59. Freshly perfused adult worms were fixed for whole mount assays with paraformaldehyde and incubated with primary antibodies and Alexa Fluor secondary antibodies before imaging on confocal microscope. (**A**,**D)** Anti-Sm29 antibodies confirmed the presence of Sm29 (in green) at the surface of the tegument as previously described^13^. (**B**,**E)** Anti-CD59 antibodies showed that CD59 (in red) was also found at the surface of the worms. (**C**,**F)** Merge of anti-Sm29 (in green) and anti-CD59 (in red) revealed that Sm29 and CD59 co-localized at the tegument surface of adult worms (in yellow).

Next, to determine whether Sm29, a tegument surface antigen shown to be a potent vaccine candidate^13^, interacts with CD59, a potent inhibitor of the MAC, we first looked for the presence of CD59 in two stages of schistosome development, cercariae-derived schistosomula and freshly perfused adult worms. Interestingly, lysate of adult male and female worms recovered from perfused hamsters revealed the presence of CD59 by western blot, while CD59 was not found in schistosomula derived from mechanical transformation of cercariae (Fig. 5). The presence of CD59 was also observed in lysates of adult worms from perfused mice (data not shown). This observation is compatible with the fact that only adult worms were in contact with the vertebrate host and not the schistosomula suggesting strongly that CD59, recognized in the western blot, might come from the host environment. However, adult worms that were cultivated from one to seven days did not exhibit any CD59 suggesting that CD59 may have been absorbed or degraded following tegument regeneration. Sm29 is a transmembrane protein with an established role in immune evasion but the mechanism is uncharacterized. Located at the surface of schistosome tegument, Sm29 appears at the interaction interface with the vertebrate- host environment. Therefore, following our prospective predictions, we performed localization studies of the Sm29 and CD59 in freshly perfused adult worms to identify any putative interaction of Sm29 and CD59 (Fig. 6). Confocal images confirmed that Sm29 was located at the tegument surface of adult worms as previously described^13^. In the same worms, CD59 was also seen at the surface of the tegument. Interestingly, Sm29 and CD59 co-localized at the surface of the tegument at the same loci suggesting that both proteins interact together as predicted by our model.

Further experiments will be needed to characterize the direct mechanism of interaction between Sm29 and CD59 and, if confirmed, this will provide additional insight on how *S. mansoni* inhibits the MAC and additional strategies for preventing this inhibition.

### Limitations

The *S. mansoni* genome was only recently sequenced^21^, and there were fewer than 39 validated crystal structures of pathogen proteins available, with only one of these in complex with a human protein. Initial predictions rely on sequence and structure comparison to known interacting complexes, thus the lack of available protein structures in complex limits the coverage of the protocol. Additional experimental efforts will increase coverage and accuracy by identifying more *S. mansoni* and human protein interactions, more protein interactions in complex, and further characterizing the biology for comparative analysis.

Furthermore, template coverage is primarily restricted to domain- mediated interactions, although peptide-mediated interactions are also known to contribute to protein interaction networks^38^. Peptide motifs that mediate protein interactions were identified through a combination of computational and experimental methods^39,40^, and application of these motif-based methods will likely expand the coverage of host-pathogen protein interactions.

### Prediction Errors

Several factors affect the accuracy of the method. These include errors in the comparative modeling process^41^, the coarse-grained nature of the statistical potential used to assess the interface residue contacts^17^, and consideration of only interactions between individual domains that could lead to predicted interactions that were unfavorable in the context of the full-length proteins. Additionally, both *S. mansoni* and humans are eukaryotic species, which means core cellular components, such as translation machinery, metabolic enzymes, and ubiquitin-signaling components are conserved and comprise many of the initially predicted interactions.

We address the similarities in conserved structures using the Biological Context Filter to remove complexes where there was a low possibility of *in vivo* occurrence, homodimer complexes that clearly involve conserved machinery, and high frequency template domains that could indicate both conserved sequences and structures as well as sequence-structure bias due to lack of interacting template coverage. For example, *S. mansoni* has been shown to secrete chemokine binding proteins as a decoy mechanism that modulates the host immune response. These proteins would be difficult to identify and characterized using known proteomic analysis and would likely be homologous to human proteins and would introduce noise into the detection and isolation of these types of interactions^42^.

### Future Work

Computational prediction and identification of protein-protein interactions is an important aspect in the development of new vaccines and vaccine candidate antigens. A variety of approaches such as genomic proximity, gene fission/fusion, phylogenetic tree similarity, gene co-occurrence, co-localization, and co-expression, amongst others, only make sense or are currently feasible in the context of a single genome^43^. Comparative approaches offer a broad spectrum analysis of protein-protein interactions based on previous observations. Our model suggests that the integration of corollary data through methods such as NLP into computational predictions enriches both the data set and improves specificity of protein-protein interaction prediction.

Furthermore, the results of the targeted analysis used on *S. mansoni*-human protein interactions here suggest that enriched sequence and structure-based methods are an applicable approach^17,44^. This method could have several extensions, including those that identify peptide motifs^38^, sequence signatures^45^ that mediate interactions, and analysis of the genetic polymorphisms at loci encoding for the proposed interacting proteins. Additionally, enhancements to existing methodologies such as NLP and machine learning algorithms can further expand and improve analysis.

In this work, we have confirmed the validity of two putative schistosome-protein interactions using our prediction model with preliminary experimental validation. We anticipate that our model could be used for additional protein-protein interactions to identify or validate novel putative targets. It will be interesting to experimentally confirm the validity of the other predictions presented here.

### Potential impact

We developed a computational whole-genome method to predict potential host-pathogen protein interactions between *S. mansoni* and humans. Our results show seven validated predictions already experimentally characterized and highlight novel interactions involving proteins indicated as vaccine candidate antigens or potential vaccine candidate antigens. Despite limitations in *S. mansoni* structural coverage, our results demonstrate that broad-spectrum data enrichment and analysis is an effective method for protein-protein interaction prediction and highlight several potential immunization targets against *S. mansoni* and provide a list of high confidence predictions. Additionally, in the tradition of open source efforts of the biomedical scientific community, the application framework is available for download by request. In closing, we expect our method to complement experimental methods and provide insight into the basic biology of *S. mansoni*-human protein interactions.

## Materials and Methods

The initial predictions of *S. mansoni*-human were generated based on a protocol described in^18^, briefly reviewed here. First, genome-wide *S. mansoni* and human protein structure models were calculated by MODPIPE^46^, an automated software pipeline for large-scale protein structure modeling^47^. MODPIPE uses MODELLER^48^ to perform the canonical comparative modeling steps of fold assignment, target-template alignment, model construction, and model assessment. High-scoring models were deposited in MODBASE^49^, a publicly accessible database of comparative models. Next, resulting models were aligned to SCOP domain sequences, and if a model aligned to a SCOP sequence with more than 70% identity, it was assigned that SCOP domain identifier. These annotations were used as the basis for a search in PIBASE, a database of domain-domain interactions. In this search, those models assigned a SCOP domain that was part of a PIBASE interaction were structurally aligned to the conformation of that domain in the complex. In cases where a human model was aligned to one domain in a PIBASE interaction and a *S. mansoni* model was aligned to the other domain, a putative modeled complex resulted. This complex was then assessed with the MODTIDE potential, which outputs a Z-score approximating the statistical likelihood of the individual domain interface residues forming a complex across the two proteins. A detailed description of the full protocol is available in^17^. We refer to the resulting set of predictions as Initial Predictions.

### Filtering Interactions

Two sets of filters were applied to the resulting interactions. The first filter, referred to as the Network Filter was based on aspects of the modeling and scoring process. The second filter, referred to as the Biological Context Filter, was based on the stages of the life cycle and tissue pairs (Fig. 1).

### Application of Network Filters

Predictions based on templates used for more than 1% of the total number of *S. mansoni* and human interactions were considered promiscuous and removed. 242,677 (45.9%) (Table 1) interactions met this criterion due to the overall similarity in eukaryotic organisms for network level machinery^50^ and to the lack of known structure information for *S. mansoni* proteins. High confidence interactions were isolated based on previous work demonstrating an optimal statistical potential Z-score threshold of −1.7, which gave true-positive and false-positive rates of 97% and 3%, respectively^18^. The homodimer complex filter removed predicted interactions based on template complexes formed by protein domains from the same SCOP family excluding highly conserved eukaryotic pathways. These predictions primarily consisted of multimeric enzyme complexes formed by host and pathogen proteins, as well as core cellular components such as ribosome subunits, proteasome subunits, and core cellular components^18^. In total, 143,065 homo-dimer complexes were removed from the filter set based on this criteria (Table 1).

### Application of Biological Context Filters

Interactions that pass the Network filtering are then filtered for biological context using the following methods.

#### Natural Language Processing (NLP) & Enrichment Filters

*S. mansoni* Protein Annotation: In preparation for applying the Life Cycle/Tissue Filter, a Natural Language Processing (NLP) protocol was created to automatically identify from the literature which *S. mansoni* proteins were expressed in different pathogen life cycle stages. *S. mansoni* protein database identifiers and their amino acid sequences were extracted from the GeneDB^20^, National Center for Biotechnology Information [NCBI], TIGR^51^, and Uniprot/TrEMBL databases^52^. A literature search identified experiments indicating proteins expressed in different *S. mansoni* life cycle stages and categorized each literature reference into corresponding life cycle stages. All literature was then mined using NLP to derive accessions and context information. Accessions were derived from the text with regular expression searches corresponding to the specifications of the database (for example, a word in the text matching the regular expression form [A-Z][0–9]5 indicates a Uniprot Accession).

Thus, for each paper, a list of protein accessions was obtained. All protein accessions were then mapped by comparing sequences to Smp accession, Uniprot accession, and NCBI accession, in that order of priority. Thus, the final result of NLP processing was a list of all accessions of proteins expressed in life cycle stages of *S. mansoni*. *S. mansoni* protein sequence data from these initial interactions were enriched from biological annotation obtained from MODBASE^49^, GeneDb^20^, NCBI, Uniprot^52^, and primary reference in literature. The annotations included protein names, links to referenced resources, and any available functional annotation.

Human Protein Annotation: Human proteins were annotated for tissue expression (GNF Tissue Atlas)^53^, known expression on cell surface, and known immune system involvement (ENSEMBL)^54^. Functional annotation for each protein was obtained from Gene Ontology Annotation (GOA)^55^. Human protein sequences were correlated with predicted interacting sequences to determine involvement^17^.

#### Life Cycle Stage/Tissue Filter

Next, the Biological Context Filter was applied to *S. mansoni* and human protein interactions in the four life cycle stages associated with pathogenesis and infections in humans. *S. mansoni* proteins were filtered by life cycle stage, known expression and excretion, using NLP and database annotation. An interaction had to be present in the host tissue associated with the specific stage of pathogenesis and that *S. mansoni* life cycle stage to be included in the resulting interactions. The following life cycle stage and tissue pairs were applied to filter interactions: (1) cercariae proteins and human proteins expressed in skin, (2) schistosomula proteins and human proteins expressed in skin, lungs, bronchial, liver, endothelial cells, immune cells, red blood cells, blood, T-cells, early erythroid cells, Natural Killer (NK) cells, myeloid cells, and B-cells, (3) adult *S. mansoni* and human proteins expressed in liver, endothelial cells, immune cells, red blood cells, blood, T-cells, early erythroid cells, NK cells, myeloid cells, and B-cells, and (4) eggs and human proteins expressed in liver, endothelial cells, immune cells, red blood cells, blood, T-cells, early erythroid cells, NK cells, myeloid cells, and B-cells.

#### Targeted Filter

The final step in the Biological Context Filter uses a targeted post process analysis based on NLP and database annotations using two additional data mining steps. For each of the interacting protein complex pairs, three parameters were analyzed: pairwise expression in both known human tissue target and *S. mansoni* life cycle stage as indicated by the Life Cycle/Tissue Filter, expression or involvement in known human immunogenic responses, and *S. mansoni* protein expression or involvement with human proteins targeted by other parasites.

Parameters for additional data mining in the target analysis include the following criteria: investigator-selected proteins of interest and NLP derived key terms that were used to target annotation data in protein names and functional annotation (Uniprot^52^, GeneDB^20^, Gene Ontology [GO]^56^). Proteins selected as targets were assigned weights composed of two factors: (1) an average weight of number of citations across all references to the number of actual references used and (2) an investigator-assigned rank (1–3) based on significance and scope of primary reference/experiments of NLP sources. The names of proteins and functional annotation were mined for the weighted key terms. All investigator-selected proteins of interest were presumed to pass filter criteria and the remaining interactions were ordered based on key term weights, rank, and Z score.

### Assessments

Predictions were benchmarked against confirmed *S. mansoni*-human interactions, which were compiled from the literature. Prospective interactions were assessed using vaccine candidate antigens and hypothesized vaccine candidate antigens where interactions have not been confirmed although several potential human protein binders have been experimentally identified (Table 4). Orthogonal biological information implemented in the filters provided significant enrichment of observed interactions (97% of predicted complexes were enriched). The number of protein pairs was reduced by about three orders of magnitude and assessment against previously characterized interactions (63% of known interactions predicted) suggests the method was applicable for genome-wide predictions of protein complexes.

### Code availability

Custom code used for NLP enrichment and assessment are available upon request to the corresponding author.

### Animals and parasites

A Puerto Rican isolate of *S. mansoni* was maintained routinely by passage through *Biomphalaria glabrata* snails and 4–6 weeks old female Golden Syrian hamsters as intermediate and definitive hosts, respectively. Schistosomula were obtained by mechanical method from cercariae and adult schistosomes were collected by portal perfusion from infected hamsters as previously described^57,58^. The protocols of maintenance and handling of hamsters have been performed in accordance with the United States Public Health Service Policy on Humane Care and Use of Laboratory Animals, and the Animal Welfare Act and Regulations and have been approved by the Institutional Animal Care and Use Committee at the University of California San Francisco (Approval AN107779).

### Immunolocalization of Sm29 and CD59 in adult worms of *S. mansoni*

Adult worms freshly recovered from perfused hamsters were used in whole mount assays for confocal microscopy studies according to^13^ with minor modifications. Worms were washed twice with PBS then fixed for two hours in 4% paraformaldehyde in PBS at room temperature under agitation prior to permeabilization (0.1% Triton X-100, 0.1% sodium citrate) for 10 minutes on ice. Samples were blocked for two hours at 4 °C under agitation in blocking buffer (PBS, 1% BSA, 0.1% Triton X-100). Blocked parasites were incubated with anti-Sm29 mouse serum (gift from Sergio C. Oliveira)^13^ and anti-CD59 (R79) rabbit polyclonal antibody (Santa Cruz Biotechnology), both diluted 1:100 in the blocking buffer overnight at 4 °C under agitation, followed by six washes with 0.05% Tween 20 in PBS. Samples were incubated with secondary anti-mouse (Invitrogen, Alexa Fluor 488) and anti-rabbit (Invitrogen, Alexa Fluor 633), both diluted 1:800, during 4 hours at 4 °C under agitation, and washed extensively prior to mounting in 90% glycerol and 10% 1 M Tris pH 8.4. Images were taken using the Zeiss LSM 510 confocal microscope.

### Western blot analysis

Adult worms and schistosomula were ground with mortar and pestle in 1x LDS buffer (Invitrogen) and separated using 12% NuPage bis-tris precast polyacrylamide gels (Invitrogen). Proteins were transferred to a PVDF membrane (BioRad) and blocked 1 hour at room temperature with 1% BSA in TBS (Tris buffered saline, pH 7.6). The membrane was then probed with a mouse anti-CD59 antibody (Santa Cruz Biotechnology) diluted 1:1,000 in TBST overnight at 4 °C. The membranes were washed three times and probed two hours with peroxidase-conjugated anti-mouse IgG antibody (GE healthcare) diluted 1:10,000 in 3% BSA in TBST. After three washes with TBST, proteins were visualized using SuperSignal West Pico Chemiluminescent Substrate (Thermo Scientific).

### Cercarial elastase (SmCE) activity assay

The schistosome cercarial elastase SmCE was purified from cercarial secretions as previously described^59^. SmCE was incubated with or without elafin (Sino Biological Inc) at 54 μM and 177 μM in 100 μL of assay buffer (100 mM Tris, pH 8) for 1 hour at room temperature. Protease activity of SmCE was then assayed using an activity-based probe, fluorophosphanate rhodamine (FP-rhodamine) (kindly provided by Ben Cravatt, Scripps Research Institute) added to 0.5 μM final concentration in samples for 10 minutes. Samples were loaded in 12% NuPage bis-tris precast polyacrylamide gels (Invitrogen) and bands corresponding to active SmCE were visualized using Typhoon Trio (GE Healthcare Life Sciences). In addition, to evaluate whether elafin is a substrate of SmCE, samples as prepared above were loaded in 12% NuPage bis-tris precast polyacrylamide gels (Invitrogen) and proteins were visualized with Coomassie blue staining. As controls, pancreatic elastase (PE) (Sigma) was assayed with or without elafin at 54 μM and ecotin (Sino Biological Inc) was used as an inhibitor of SmCE at 54 μM and 177 μM.

## Electronic supplementary material

Supplemental Interactions

## Data Availability

The custom NLP algorithm referenced here is publicly available by request. Additional results are available as supplemental material.
