# Supplementary material for: Predictions of novel Schistosoma mansoni - human protein interactions consistent with experimental data
Source: Sci Rep. 2018 Aug 30;8:13092. doi: 10.1038/s41598-018-31272-1 (PMC6117258; doi:10.1038/s41598-018-31272-1)
Supplement: Supplementary file 1 — Supplemental Interactions [file 41598_2018_31272_MOESM1_ESM.pdf]

# Predictions of novel *Schistosoma mansoni* - human protein interactions consistent with experimental data

J. White Bear[1,2,6,&,*], Thavy Long [3,4,5,*], Danielle Skinner [4], James H. McKerrow [3,4]

**1 Department of Bioengineering and Therapeutic Sciences, Department of Pharmaceutical Chemistry, and California Institute for Quantitative Biosciences, University of California, San Francisco, CA 94158**

**2 Graduate Group in Bioinformatics, University of California, San Francisco, CA 94158, USA**

**3 Department of Pathology and Sandler Center for Basic Research in Parasitic Diseases, University of California at San Francisco, San Francisco, California 94158, USA**

**4 Skaggs School of Pharmacy and Pharmaceutical Sciences, University of California San Diego 9500 Gilman Dr, La Jolla, CA 92093**

**5 (Current Address) INRA - InTheRes - UMR 1436, Equipe Transporteurs Membranaires et Résistance; 180, Chemin de Tournefeuille, Toulouse, France**

**6 (Current Address) MIT Lincoln Laboratory 244 Wood St. Lexington, MA US**

**& Materials & Correspondence: J. White Bear Email: jwbear15@gmail.com**

**∗ These authors contributed equally to this work.**

# Supplemental Information 1: Cercarial Elastase Interactions

## *Cercarial Elastase Interactions*

| S. mansoni | Human | Human Protein |
|---|---|---|
| Smp_112090 | 148613878 gi | acrosin precursor [Homo sapiens] |
| SMP_006510 | 148613878 gi | acrosin precursor [Homo sapiens],Acrosin precursor (EC 3.4.21.10) [Contains: Ac |
| SMP_119130 | 38504669 gi | serine (or cysteine) proteinase inhibitor, clade B (ovalbumin), member 8 [Homo |
| SMP_006510 | 38504669 gi | serine (or cysteine) proteinase inhibitor, clade B (ovalbumin), member 8 [Homo |
| SMP_006510 | 21359871 gi | SPARC-like 1 [Homo sapiens] |
| Smp_006520 | 134268640 gi | tectorin alpha precursor [Homo sapiens] |
| SMP_006510 | 134268640 gi | tectorin alpha precursor [Homo sapiens], |
| SMP_112090 | 134268640 gi | tectorin alpha precursor [Homo sapiens], |
| Smp_119130 | 21614538 gi | (O43464) Splice isoform 2 of O43464 |
| SMP_006510 | 31563528 gi | (O95925) Splice isoform 2 of O95925,AF286369_1 eppin-2,serine protease inhibitor |
| Smp_112090 | 58533163 gi | (P14210) Splice isoform 2 of P14210 |
| SMP_006510 | 58533163 gi | (P14210) Splice isoform 2 of P14210,Hepatocyte growth factor, heavy chain precur |
| Smp_112090 | 58533170 gi | (P14210) Splice isoform 3 of P14210 |
| SMP_006510 | 58533170 gi | (P14210) Splice isoform 3 of P14210,HEPATOCYTE GROWTH FACTOR |
| Smp_006520 | 5453652 gi | (P19883) Splice isoform 2 of P19883 |
| SMP_006510 | 5453652 gi | (P19883) Splice isoform 2 of P19883,follistatin precursor |
| Smp_006510 | 21536452 gi | (P35030) Splice isoform C of P35030,(P35030) Splice isoform C of P35030,Protease |
| Smp_006510 | 9665236 gi | (Q9UKR0) Splice isoform 2 of Q9UKR0,(Q9UKR0) Splice isoform 2 of Q9UKR0,kallikre |
| Smp_006510 | 153791781 gi | 48 kDa protein [Homo sapiens],48 kDa protein [Homo sapiens],Uncharacterized prot |
| Smp_006520 | 42544239 gi | Adipsin/complement factor D precursor (EC 3.4.21.46) (ComplementDE factor D, p |
| SMP_006510 | 42544239 gi | Adipsin/complement factor D precursor (EC 3.4.21.46) (ComplementDE factor D, p |
| Smp_119130 | 89142741 gi | AF243527_5 serine protease |
| SMP_006510 | 89142741 gi | AF243527_5 serine protease,serine protease prostase,Q9Y5K2_chr19:56102005-561058 |
| SMP_006510 | 54873613 gi | Agrin |
| SMP_006510 | 55743100 gi | alpha 3 type VI collagen isoform 2 precursor [Homo sapiens] |
| SMP_006510 | 55743104 gi | alpha 3 type VI collagen isoform 4 precursor [Homo sapiens]. |
| SMP_006510 | 4502067 gi | Alpha-1-microglobulin/bikunin (Growth-inhibiting protein 19),,AMBP protein precu |
| SMP_112090 | 115583663 gi | Alpha-2-antiplasmin precursor (Alpha-2-plasmin inhibitor) (Alpha-2-PI)DE (Alph |
| SMP_006510 | 115583663 gi | Alpha-2-antiplasmin precursor (Alpha-2-plasmin inhibitor) (Alpha-2-PI)DE (Alph |
| Smp_112090 | 41406055 gi | Amyloid beta (A4) protein (Peptidase nexin-II, Alzheimer disease) |
| SMP_006510 | 41406055 gi | Amyloid beta (A4) protein (Peptidase nexin-II, Alzheimer disease),Amyloid beta A |
| SMP_119130 | 4502147 gi | Amyloid-like protein 2 precursor (Amyloid protein homolog) (APPH)DE (CDEI box- |

# Supplemental Information 1: Cercarial Elastase Interactions



| | | |
|---|---|---|
| SMP_006510 | 4502147 gi | Amyloid-like protein 2 precursor (Amyloid protein homolog) (APPH)DE (CDEI box- |
| SMP_119130 | 4557287 gi | Angiotensinogen (Serine (Or cysteine) proteinase inhibitor, clade ADE (Alpha-1 |
| SMP_006510 | 4557287 gi | Angiotensinogen (Serine (Or cysteine) proteinase inhibitor, clade ADE (Alpha-1 |
| SMP_006510 | 4507065 gi | Antileukoproteinase 1 precursor (ALP) (HUSI-1) (Seminal proteinaseDE inhibitor |
| Smp_119130 | 4507065 gi | Antileukoproteinase 1 precursor (ALP) (HUSI-1) (Seminal proteinaseDE inhibitor |
| Smp_006510 | 13346502 gi | Apolipoprotein (A) related gene C (Lipoprotein, Lp(A)-like 2,),Apolipoprotein (A |
| Smp_006520 | 41350214 gi | Asporin (LRR class 1) |
| Smp_112090 | 29244926 gi | atrial natriuretic peptide-converting enzyme [Homo sapiens] |
| Smp_006510 | 11342670 gi | Azurocidin 1, preproprotein,Azurocidin 1, preproprotein,Azurocidin precursor (Ca |
| Smp_006510 | 11545839 gi | Brain-specific serine protease-4 (Serine protease PRSS22),Brain-specific serine |
| Smp_006510 | 22208982 gi | Breast normal epithelial cell associated serine protease (KallikreinDE 10),Bre |
| Smp_006510 | 62526043 gi | Caldecrin precursor (EC 3.4.21.2) (Chymotrypsin C),Caldecrin precursor (EC 3.4.2 |
| Smp_006520 | 4502997 gi | Carboxypeptidase A1 precursor (EC 3.4.17.1) |
| SMP_119130 | 4502997 gi | Carboxypeptidase A1 precursor (EC 3.4.17.1),Carboxypeptidase A1 (Pancreatic),Car |
| Smp_006520 | 4502999 gi | CARBOXYPEPTIDASE A2 PRECURSOR (EC 3.4.17.15) |
| Smp_119130 | 126273559 gi | carboxypeptidase B2 (plasma, carboxypeptidase U), isoform CRA_a |
| SMP_006510 | 22202611 gi | Carboxypeptidase D,,Similar to carboxypeptidase D (CPD protein),Carboxypeptidase |
| Smp_112090 | 4503149 gi | Cathepsin G precursor (EC 3.4.21.20) (CG) |
| SMP_006510 | 4503149 gi | Cathepsin G precursor (EC 3.4.21.20) (CG),Cathepsin G precursor (EC 3.4.21.20) ( |
| SMP_006510 | 58218970 gi | cDNA FLJ55455, highly similar to Polyserase-2 (EC 3.4.21.-) |
| Smp_006510 | 24308541 gi | CDNA FLJ90724 fis, clone PLACE1009279, weakly similar toDE Physiologically act |
| SMP_006510 | 10190748 gi | CEGP1 protein |
| Smp_112090 | 4502907 gi | Chymase precursor (EC 3.4.21.39) (Mast cell protease I) |
| SMP_006510 | 4502907 gi | Chymase precursor (EC 3.4.21.39) (Mast cell protease I),Chymase precursor (EC 3. |
| Smp_006510 | 4503137 gi | Chymotrypsin-like protease CTRL-1 precursor (EC 3.4.21.-),Chymotrypsin-like prot |
| Smp_006520 | 118498341 gi | chymotrypsinogen B precursor [Homo sapiens] |
| Smp_112090 | 118498350 gi | chymotrypsinogen B2 precursor [Homo sapiens] |
| Smp_006510 | 4503649 gi | Coagulation factor IX (Plasma thromboplastic component, ChristmasDE disease, h |
| Smp_119130 | 10518503 gi | Coagulation factor VII (Serum prothrombin conversion accelerator) |
| SMP_006510 | 10518503 gi | Coagulation factor VII (Serum prothrombin conversion accelerator),Coagulation fa |
| Smp_119130 | 4503645 gi | Coagulation factor VII (Serum prothrombin conversion accelerator)DE (FVII coag |
| SMP_006510 | 4503645 gi | Coagulation factor VII (Serum prothrombin conversion accelerator)DE (FVII coag |
| Smp_006510 | 4503625 gi | Coagulation factor X,Coagulation factor X,Coagulation factor X precursor (EC 3.4 |
| SMP_006510 | 145275213 gi | coagulation factor XII precursor [Homo sapiens] |
| Smp_006520 | 4502961 gi | COLLAGEN ALPHA 1(VII) CHAIN PRECURSOR (LONG-CHAIN COLLAGEN) (LCDE COLLAGEN) |
| SMP_006510 | 4502961 gi | COLLAGEN ALPHA 1(VII) CHAIN PRECURSOR (LONG-CHAIN COLLAGEN) (LCDE COLLAGEN),Co |

# Supplemental Information 1: Cercarial Elastase Interactions



| | | |
|---|---|---|
| SMP_006510 | 55743098 gi | collagen alpha-3(VI) chain isoform 1 precursor [Homo sapiens] |
| SMP_006510 | 55743102 gi | collagen alpha-3(VI) chain isoform 3 precursor [Homo sapiens] |
| SMP_006510 | 55743106 gi | collagen alpha-3(VI) chain isoform 5 precursor [Homo sapiens] |
| Smp_119130 | 93141047 gi | Collagen type XII alpha 1 |
| Smp_006510 | 7706083 gi | Complement C1r-like proteinase,Complement C1r-like proteinase,Q9NZP8_chr12:71401 |
| Smp_006510 | 4502495 gi | Complement C1s subcomponent precursor (EC 3.4.21.42) (C1 esterase)DE [Contains |
| Smp_112090 | 119392081 gi | Complement factor I precursor (EC 3.4.21.45) (C3B/C4B inactivator)[Contains: Com |
| SMP_006510 | 119392081 gi | Complement factor I precursor (EC 3.4.21.45) (C3B/C4B inactivator)[Contains: Com |
| Smp_006510 | 21264359 gi | Complement factor MASP-3 (Mannan-binding lectin serine protease 1,DE isoform 2 |
| Smp_119130 | 92110053 gi | CUB and Sushi multiple domains 2 |
| Smp_119130 | 10092639 gi | Cysteine-rich motor neuron 1 |
| SMP_112090 | 10092639 gi | Cysteine-rich motor neuron 1,Q9NZV1_chr2:36557971-36750376_G9V Cysteine-rich rep |
| SMP_006510 | 10092639 gi | Cysteine-rich motor neuron 1,Q9NZV1_chr2:36557971-36750376_G9V Cysteine-rich rep |
| Smp_006520 | 110735443 gi | DJ894D12.3 (Delta-like 1 (Mouse) homolog) |
| SMP_119130 | 110735443 gi | DJ894D12.3 (Delta-like 1 (Mouse) homolog),O00548_chr6:170448474-170455736_R502 D |
| SMP_006510 | 4505787 gi | Elafin precursor (Elastase-specific inhibitor) (ESI) (Skin-derivedDE antileuko |
| Smp_006520 | 4505787 gi | Elafin precursor (Elastase-specific inhibitor) (ESI) (Skin-derivedDE antileuko |
| Smp_006520 | 58331209 gi | Elastase 1, pancreatic |
| SMP_006510 | 58331209 gi | Elastase 1, pancreatic,Elastase-1 precursor (EC 3.4.21.36), |
| Smp_006510 | 4503549 gi | Elastase 2, neutrophil,Elastase 2, neutrophil,Leukocyte elastase precursor (EC 3 |
| Smp_112090 | 58331211 gi | Elastase 2B, preproprotein |
| SMP_006510 | 58331211 gi | Elastase 2B, preproprotein,Elastase 2B,Elastase 2B.,Pancreatic elastase IIB |
| Smp_112090 | 6679625 gi | Elastase 3B, pancreatic |
| SMP_006510 | 6679625 gi | Elastase 3B, pancreatic, |
| Smp_006510 | 15559207 gi | Elastase-2A precursor (EC 3.4.21.71),Elastase-2A precursor (EC 3.4.21.71),Elasta |
| Smp_006510 | 58331214 gi | Elastase-3A precursor (EC 3.4.21.70) (Elastase IIIA) (Protease E),Elastase-3A pr |
| SMP_006510 | 50659100 gi | ELGC699 |
| Smp_006510 | 4506151 gi | Enteropeptidase precursor (EC 3.4.21.9) (Enterokinase) (Serineprotease 7) [Conta |
| Smp_112090 | 117956391 gi | EOS protein (Hypothetical protein) |
| SMP_006510 | 117956391 gi | EOS protein (Hypothetical protein),PRSS33 protein (Protease, serine, 33),PRSS33 |
| Smp_006520 | 21614533 gi | eosinophil serine protease 1 splicing variant |
| SMP_006510 | 21614533 gi | eosinophil serine protease 1 splicing variant,(Q9Y6M0) Splice isoform 3 of Q9Y6M |
| Smp_006510 | 12408682 gi | Epididymal sperm-binding protein 1 (Epididymal secretory protein 12)DE (hE12), |
| SMP_119130 | 10732863 gi | Eppin precursor (Epididymal protease inhibitor) (Serine proteaseDE inhibitor-l |
| SMP_006510 | 10732863 gi | Eppin precursor (Epididymal protease inhibitor) (Serine proteaseDE inhibitor-l |
| Smp_006520 | 14211875 gi | Esophagus cancer-related gene-2 |

# Supplemental Information 1: Cercarial Elastase Interactions



| | | |
|---|---|---|
| SMP_112090 | 14211875 gi | Esophagus cancer-related gene-2,,Esophagus cancer-related gene-2 protein precurs |
| SMP_006510 | 14211875 gi | Esophagus cancer-related gene-2,,Esophagus cancer-related gene-2 protein precurs |
| Smp_112090 | 71040111 gi | Fibromodulin, |
| SMP_006510 | 5901956 gi | Follistatin-related protein 1 precursor (Follistatin-like 1), |
| SMP_119130 | 5901956 gi | Follistatin-related protein 1 precursor (Follistatin-like 1),,Follistatin-relate |
| SMP_006510 | 5031701 gi | Follistatin-related protein 3 precursor (Follistatin-like 3)DE (Follistatin-re |
| SMP_006510 | 54792136 gi | follistatin-related protein 4 precursor [Homo sapiens] |
| SMP_006510 | 50363221 gi | Full-length cDNA clone CS0DE007YP21 of Placenta of Homo sapiensDE (human) (Ful |
| Smp_112090 | 5453676 gi | Granzyme A precursor (EC 3.4.21.78) (Cytotoxic T-lymphocyte proteinase1) (Hanukk |
| SMP_006510 | 5453676 gi | Granzyme A precursor (EC 3.4.21.78) (Cytotoxic T-lymphocyte proteinase1) (Hanukk |
| Smp_112090 | 4758494 gi | Granzyme B precursor (EC 3.4.21.79) (T-cell serine protease 1-3E)(Cytotoxic T-ly |
| SMP_006510 | 4758494 gi | Granzyme B precursor (EC 3.4.21.79) (T-cell serine protease 1-3E)(Cytotoxic T-ly |
| Smp_006510 | 15529990 gi | Granzyme H precursor (EC 3.4.21.-) (Cytotoxic T-lymphocyte proteinase)DE (Cath |
| Smp_006510 | 4504235 gi | Granzyme K precursor (EC 3.4.21.-) (Granzyme-3) (NK-tryptase-2) (NK-DE TRYP-2) |
| Smp_112090 | 4885369 gi | Granzyme M precursor (EC 3.4.21.-) (Met-ase) (Natural killer cellgranular protea |
| SMP_006510 | 4885369 gi | Granzyme M precursor (EC 3.4.21.-) (Met-ase) (Natural killer cellgranular protea |
| Smp_006510 | 4826762 gi | Haptoglobin,Haptoglobin,P00738_chr16:71864828-71871062_S243P_G248R_T372A_D397H H |
| SMP_006510 | 45580723 gi | haptoglobin-related protein precursor [Homo sapiens] |
| SMP_006510 | 169217813 gi | hCG2002962, trypsin |
| | temporary entı | hCG2002962" |
| Smp_119130 | 126012571 gi | Heparan sulfate proteoglycan 2 (Perlecan) |
| SMP_006520 | 73858566 gi | Heparin cofactor 2 precursor (Heparin cofactor II) (HC-II) (ProteaseDE inhibit |
| SMP_006510 | 73858566 gi | Heparin cofactor 2 precursor (Heparin cofactor II) (HC-II) (ProteaseDE inhibit |
| Smp_006510 | 4504383 gi | Hepatocyte growth factor activator,Hepatocyte growth factor activator,Hepatocyte |
| Smp_006510 | 74027265 gi | hepatocyte growth factor activator inhibitor,hepatocyte growth factor activator |
| Smp_112090 | 33859835 gi | Hepatocyte growth factor precursor (Scatter factor) (SF)DE (Hepatopoeitin A) [ |
| SMP_006510 | 33859835 gi | Hepatocyte growth factor precursor (Scatter factor) (SF)DE (Hepatopoeitin A) [ |
| Smp_112090 | 4758502 gi | HGF activator like protein (Hyaluronan binding protein 2) |
| SMP_006510 | 4758502 gi | HGF activator like protein (Hyaluronan binding protein 2),Q14520_chr10:114977468 |
| Smp_119130 | 26787982 gi | Homo sapiens clone 23888 mRNA sequence (IL15RA protein) |
| SMP_006510 | 93141001 gi | HSAJ1454,AAQ89332_chr4:168351965-168851214_R97H_I112V,,HSAJ1454 |
| SMP_112090 | 54607080 gi | Hypothetical 47.4 kDa protein,Unknown (protein for MGC:21282,Carboxypeptidase B |
| SMP_006520 | 21361302 gi | Hypothetical 48.5 kDa protein, serine (or cysteine) proteinase inhibitor, clade |
| SMP_006510 | 21361302 gi | Hypothetical 48.5 kDa protein, serine (or cysteine) proteinase inhibitor, clade |
| Smp_119130 | 157738643 gi | Hypothetical protein DKFZp434G0625 |
| SMP_119130 | 4507171 gi | Hypothetical protein DKFZp468G1528,P09486_chr5:151071645-151084259_P19S_T128I_R1 |

# Supplemental Information 1: Cercarial Elastase Interactions



| | | |
|---|---|---|
| SMP_006510 | 4507171 gi | Hypothetical protein DKFZp468G1528,P09486_chr5:151071645-151084259_P19S_T128I_R1 |
| Smp_006510 | 38505209 gi | Hypothetical protein DKFZp686A06175,Hypothetical protein DKFZp686A06175, |
| SMP_006510 | 38679890 gi | Hypothetical protein FLJ46651 (Organic anion transporter OATP-DE M1) |
| Smp_112090 | 4506115 gi | Hypothetical protein PROC |
| SMP_006510 | 4506115 gi | Hypothetical protein PROC,Vitamin K-dependent protein C precursor (EC 3.4.21.69) |
| Smp_006520 | 5454114 gi | Hypothetical protein TFPI |
| SMP_119130 | 5454114 gi | Hypothetical protein TFPI,P10646_chr2:188534209-188571038_V292M TISSUE FACTOR PA |
| SMP_006510 | 5454114 gi | Hypothetical protein TFPI,P10646_chr2:188534209-188571038_V292M TISSUE FACTOR PA |
| SMP_112090 | 61743916 gi | Hypothetical protein,CPA4 protein,Carboxypeptidase A4 (Carboxypeptidase A4, isof |
| SMP_006510 | 122937420 gi | inactive serine protease 54 precursor [Homo sapiens] |
| SMP_112090 | 4504619 gi | Insulin-like growth factor-binding protein 7 precursor (IGFBP-7) (IBP-DE 7) (I |
| SMP_006510 | 4504619 gi | Insulin-like growth factor-binding protein 7 precursor (IGFBP-7) (IBP-DE 7) (I |
| SMP_006520 | 20127446 gi | Integrin beta-5 precursor,P18084_chr3:125803374-125926748_T193A_L333F_L428V_R431 |
| Smp_119130 | 4504649 gi | Interleukin 15 receptor, alpha |
| Smp_119130 | 117306176 gi | Kallikrein 5 splice variant 2 (Kallikrein 5) (Kallikrein 5 spliceDE variant 1) |
| SMP_006510 | 117306176 gi | Kallikrein 5 splice variant 2 (Kallikrein 5) (Kallikrein 5 spliceDE variant 1) |
| Smp_112090 | 61744424 gi | Kallikrein 6 |
| SMP_006510 | 61744424 gi | Kallikrein 6,Kallikrein 6.,Kallikrein-6 precursor (EC 3.4.21.-) (Protease M) (Ne |
| Smp_006510 | 29366812 gi | Kallikrein 9,Kallikrein 9,Kallikrein 9.,Kallikrein-9 precursor (EC 3.4.21.-) (Ka |
| Smp_006520 | 5803199 gi | Kallikrein-11 precursor (EC 3.4.21.-) (Hippostasin) (Trypsin-likeDE protease) |
| SMP_006510 | 5803199 gi | Kallikrein-11 precursor (EC 3.4.21.-) (Hippostasin) (Trypsin-likeDE protease), |
| Smp_112090 | 22208987 gi | Kallikrein-12 precursor (EC 3.4.21.-) (Kallikrein-like protein 5)DE (KLK-L5) |
| SMP_006510 | 22208987 gi | Kallikrein-12 precursor (EC 3.4.21.-) (Kallikrein-like protein 5)DE (KLK-L5),K |
| Smp_112090 | 11496281 gi | Kallikrein-13 precursor (EC 3.4.21.-) (Kallikrein-like protein 4)DE (KLK-L4) |
| SMP_006510 | 11496281 gi | Kallikrein-13 precursor (EC 3.4.21.-) (Kallikrein-like protein 4)DE (KLK-L4),K |
| Smp_119130 | 20302143 gi | Kallikrein-15 precursor (EC 3.4.21.-) (ACO protease) |
| SMP_006510 | 20302143 gi | Kallikrein-15 precursor (EC 3.4.21.-) (ACO protease),Kallikrein-15 precursor (EC |
| Smp_112090 | 5031829 gi | Kallikrein-2 precursor (EC 3.4.21.35) (Tissue kallikrein-2) (GlandularDE kalli |
| SMP_006510 | 5031829 gi | Kallikrein-2 precursor (EC 3.4.21.35) (Tissue kallikrein-2) (GlandularDE kalli |
| Smp_006510 | 21327705 gi | Kallikrein-7 precursor (EC 3.4.21.-) (hK7) (Stratum corneumDE chymotryptic enz |
| Smp_006510 | 91823048 gi | Kallikrein-related peptidase 14,Kallikrein-related peptidase 14,Kallikrein-relat |
| SMP_006520 | 19923632 gi | Kazal-type serine protease inhibitor domain 1 (FKSG28) (Novel kazal-DE type se |
| SMP_006510 | 19923632 gi | Kazal-type serine protease inhibitor domain 1 (FKSG28) (Novel kazal-DE type se |
| Smp_006520 | 5901992 gi | Keratocan precursor (KTN) (Keratan sulfate proteoglycan keratocan) |
| SMP_006510 | 20302147 gi | KLK15 splice variant 3 |
| SMP_112090 | 32313599 gi | Kunitz-type protease inhibitor 1 precursor (Hepatocyte growth factorDE activat |

# Supplemental Information 1: Cercarial Elastase Interactions



| | | |
|---|---|---|
| SMP_006510 | 32313599 gi | Kunitz-type protease inhibitor 1 precursor (Hepatocyte growth factorDE activat |
| Smp_119130 | 38045910 gi | Laminin alpha 3 splice variant b1 |
| Smp_006510 | 116292750 gi | Lipoprotein, Lp(A),Lipoprotein, Lp(A),Lipoprotein, Lp(A).,OTTHUMP00000017543 |
| SMP_006520 | 93102379 gi | Low-density lipoprotein receptor-related protein 1B precursor (Low-DE density |
| Smp_006510 | 93102379 gi | Low-density lipoprotein receptor-related protein 1B precursor (Low-DE density |
| Smp_112090 | 31543212 gi | Macrophage stimulating 1 (Hepatocyte growth factor-like) |
| SMP_006510 | 31543212 gi | Macrophage stimulating 1 (Hepatocyte growth factor-like),Macrophage stimulating |
| SMP_006510 | 21264357 gi | mannan-binding lectin serine protease 1 isoform 1 precursor [Homo sapiens] |
| Smp_112090 | 21264363 gi | Mannan-binding lectin serine protease 2 |
| SMP_006510 | 21264363 gi | Mannan-binding lectin serine protease 2,OTTHUMP00000044363, |
| Smp_112090 | 34098976 gi | Marapsin 2 precursor |
| SMP_006510 | 34098976 gi | Marapsin 2 precursor,Marapsin 2 precursor (Marapsin 2), |
| Smp_006510 | 4503001 gi | Mast cell carboxypeptidase A precursor (EC 3.4.17.1) (MC-CPA)(Carboxypeptidase A |
| SMP_006510 | 68508970 gi | mast cell tryptase beta III |
| SMP_006510 | 151301154 gi | mucin-6 precursor [Homo sapiens] |
| SMP_006510 | 28212222 gi | Multivalent protease inhibitor protein, |
| SMP_006520 | 28212222 gi | Multivalent protease inhibitor protein,,Multivalent protease inhibitor protein |
| Smp_006510 | 71361688 gi | Myeloblastin precursor (EC 3.4.21.76) (Leukocyte proteinase 3) (PR-3)DE (PR3) |
| Smp_112090 | 6005844 gi | Neuropsin precursor (EC 3.4.21.-) (NP) (Kallikrein-8) (Ovasin) (SerineDE prote |
| SMP_006510 | 6005844 gi | Neuropsin precursor (EC 3.4.21.-) (NP) (Kallikrein-8) (Ovasin) (SerineDE prote |
| Smp_006510 | 21464127 gi | neuropsin type2,neuropsin type2,(O60259) Splice isoform 2 of O60259 |
| Smp_006520 | 4506143 gi | Neurotrypsin precursor (EC 3.4.21.-) (Serine protease 12) (Motopsin)(Leydin) |
| SMP_006510 | 4506143 gi | Neurotrypsin precursor (EC 3.4.21.-) (Serine protease 12) (Motopsin)(Leydin),Neu |
| Smp_119130 | 55770876 gi | Notch4 |
| Smp_006520 | 122937343 gi | Novel protein similar to mouse Jedi soluble isoform 736 protein |
| SMP_006510 | 19923780 gi | organic anion transport polypeptide 2 |
| SMP_006510 | 7019531 gi | Organic anion transporter OATP-D |
| SMP_112090 | 39777594 gi | Organic anion transporter OATP-E (Colon organic anion transporter)DE (Organic |
| SMP_006510 | 39777594 gi | Organic anion transporter OATP-E (Colon organic anion transporter)DE (Organic |
| Smp_006510 | 4506121 gi | OTTHUMP00000018738,OTTHUMP00000018738,Protein Z, vitamin K-dependent plasma glyc |
| SMP_119130 | 4506121 gi | OTTHUMP00000018738,Protein Z, vitamin K-dependent plasma glycoprotein,P22891_chr |
| Smp_112090 | 110815798 gi | ovochymase-1 precursor [Homo sapiens] |
| Smp_112090 | 148231605 gi | ovochymase-2 precursor [Homo sapiens] |
| SMP_006520 | 45505132 gi | Pancreatic secretory trypsin inhibitor precursor (Tumor-associatedDE trypsin i |
| Smp_112090 | 45505132 gi | Pancreatic secretory trypsin inhibitor precursor (Tumor-associatedDE trypsin i |
| SMP_006510 | 45505132 gi | Pancreatic secretory trypsin inhibitor precursor (Tumor-associatedDE trypsin i |

# Supplemental Information 1: Cercarial Elastase Interactions



| | | |
|---|---|---|
| SMP_006510 | 145309328 gi | papilin precursor [Homo sapiens] |
| SMP_112090 | 20336242 gi | PC1,,PC1 |
| Smp_119130 | 126273569 gi | Plasma carboxypeptidase B2, isoform a preproprotein (CarboxypeptidaseDE B2) (P |
| Smp_006520 | 4505881 gi | Plasminogen |
| SMP_006510 | 4505881 gi | Plasminogen,Plasminogen precursor (EC 3.4.21.7) [Contains: Plasmin heavy chain A |
| SMP_006510 | 87196600 gi | Polyserase-3 |
| SMP_006510 | 169172917 gi | PREDICTED: hypothetical protein LOC346702 [Homo sapiens] |
| SMP_006510 | 169163549 gi | PREDICTED: hypothetical protein LOC646960 [Homo sapiens] |
| Smp_006520 | 169217813 gi | PREDICTED: hypothetical protein [Homo sapiens]. |
| SMP_006510 | 169163744 gi | PREDICTED: similar to ACR [Homo sapiens] |
| SMP_006510 | 169173186 gi | PREDICTED: similar to hCG1643218 [Homo sapiens] |
| SMP_006510 | 169165225 gi | PREDICTED: similar to hCG1786642 [Homo sapiens] |
| PREDICTED: similar to hCG1786642" | | |
| SMP_006510 | 169164530 gi | PREDICTED: similar to hCG1818432 [Homo sapiens] |
| SMP_006510 | 169179025 gi | PREDICTED: similar to hCG31140 [Homo sapiens] |
| SMP_006510 | 169202626 gi | PREDICTED: similar to mucin 5, partial [Homo sapiens] |
| SMP_006510 | 169163027 gi | PREDICTED: similar to Putative acrosin-like protease [Homo sapiens] |
| Smp_112090 | 4504875 gi | preprokallikrein (AA -24 to 238) |
| SMP_006510 | 4504875 gi | preprokallikrein (AA -24 to 238),AF243527_1 renal kallikrein, |
| Smp_119130 | 26006869 gi | Probable protease inhibitor WAP12a precursor |
| SMP_006510 | 26006869 gi | Probable protease inhibitor WAP12a precursor,Q8IUB3_chr20:44998901-45000060_L8P_ |
| Smp_112090 | 26006867 gi | Probable protease inhibitor WAP12b precursor |
| SMP_006510 | 26006867 gi | Probable protease inhibitor WAP12b precursor, |
| Smp_112090 | 22779936 gi | Probable protease inhibitor WAP6 |
| SMP_006510 | 22779936 gi | Probable protease inhibitor WAP6, |
| Smp_112090 | 22129776 gi | Probable serine protease HTRA3 precursor (EC 3.4.21.-) (High-DE temperature re |
| SMP_006510 | 22129776 gi | Probable serine protease HTRA3 precursor (EC 3.4.21.-) (High-DE temperature re |
| Smp_112090 | 4506153 gi | Prostasin precursor (EC 3.4.21.-) (Serine protease 8) [Contains:Prostasin light |
| SMP_006510 | 4506153 gi | Prostasin precursor (EC 3.4.21.-) (Serine protease 8) [Contains:Prostasin light |
| Smp_119130 | 71834857 gi | Prostate specific antigen precursor |
| SMP_006510 | 71834857 gi | Prostate specific antigen precursor,Prostate specific antigen precursor.,Q8NCW4_ |
| Smp_119130 | 71834855 gi | prostate-specific antigen isoform 4 preproprotein [Homo sapiens] |
| Smp_112090 | 4502173 gi | Prostate-specific antigen precursor (EC 3.4.21.77) (PSA) (Kallikrein-DE 3) (Se |
| SMP_006510 | 4502173 gi | Prostate-specific antigen precursor (EC 3.4.21.77) (PSA) (Kallikrein-DE 3) (Se |
| Smp_112090 | 21618357 gi | Prostate-type hippostasin |
| SMP_006510 | 21618357 gi | Prostate-type hippostasin,Kallikrein-11 precursor (EC 3.4.21.-) (Hippostasin) (T |

# Supplemental Information 1: Cercarial Elastase Interactions



| | | |
|---|---|---|
| Smp_119130 | 68299793 gi | Protease inhibitor H |
| SMP_006510 | 68299793 gi | Protease inhibitor H,Q8N5P0_chr5:147611170-147623020_P36 Hypothetical protein, |
| Smp_006510 | 13994276 gi | Protease serine 27 precursor (EC 3.4.21.-) (Marapsin) (Pancreasin)DE (Channel- |
| SMP_006510 | 56606135 gi | protease, serine, 37 precursor [Homo sapiens] |
| SMP_119130 | 154759291 gi | Protein Z-dependent protease inhibitor precursor (PZ-dependentDE protease inhi |
| Smp_006520 | 154759291 gi | Protein Z-dependent protease inhibitor precursor (PZ-dependentDE protease inhi |
| SMP_006510 | 154759291 gi | Protein Z-dependent protease inhibitor precursor (PZ-dependentDE protease inhi |
| Smp_112090 | 4503635 gi | Prothrombin precursor (EC 3.4.21.5) (Coagulation factor II) [Contains:Activation |
| SMP_006510 | 4503635 gi | Prothrombin precursor (EC 3.4.21.5) (Coagulation factor II) [Contains:Activation |
| Smp_006510 | 11321636 gi | PUTATIVE MAST CELL MMCP-7-LIKE II TYPTASE,PUTATIVE MAST CELL MMCP-7-LIKE II TYPT |
| SMP_112090 | 16751923 gi | Putative multivalent protease inhibitor WFIKKN (WAP,DE follistatin/kazal, immu |
| SMP_006510 | 16751923 gi | Putative multivalent protease inhibitor WFIKKN (WAP,DE follistatin/kazal, immu |
| SMP_006510 | 116812593 gi | putative solute carrier organic anion transporter family member 1B7 [Homo sapien |
| SMP_006510 | 66347875 gi | R subcomponent of complement component 1 |
| Smp_006510 | 19743898 gi | Receptor tyrosine kinase-like orphan receptor 2,Receptor tyrosine kinase-like or |
| Smp_006510 | 50659098 gi | Regeneration associated muscle protease, isoform a,Regeneration associated muscl |
| SMP_006510 | 11863156 gi | Reversion-inducing-cysteine-rich protein with kazal motifs,Reversion-inducing cy |
| Smp_119130 | 32454741 gi | Rheumatoid arthritis related antigen RA-A47 |
| SMP_006510 | 32454741 gi | Rheumatoid arthritis related antigen RA-A47,P50454_chr11:75003703-75009433_L6P_A |
| SMP_006510 | 4506117 gi | S plasma protein |
| Smp_006510 | 50363237 gi | S39329_11 glandular kallikrein-1,S39329_11 glandular kallikrein-1, |
| Smp_119130 | 33598937 gi | scavenger receptor class F member 2 isoform 2 precursor [Homo sapiens] |
| Smp_119130 | 24586659 gi | Scavenger receptor class F member 2 precursor (Scavenger receptorDE expressed |
| SMP_006510 | 78190498 gi | Secreted modular calcium-binding protein 1 |
| SMP_112090 | 110225349 gi | serine (or cysteine) proteinase inhibitor, clade A (alpha-1 antiproteinase, anti |
| SMP_006510 | 110225349 gi | serine (or cysteine) proteinase inhibitor, clade A (alpha-1 antiproteinase, anti |
| Smp_006520 | 27777657 gi | Serine (Or cysteine) proteinase inhibitor, clade A (Alpha-1DE antiproteinase, |
| SMP_006510 | 27777657 gi | Serine (Or cysteine) proteinase inhibitor, clade A (Alpha-1DE antiproteinase, |
| SMP_112090 | 8393956 gi | Serine (Or cysteine) proteinase inhibitor, clade B (Ovalbumin), memberDE 13,HU |
| SMP_006510 | 8393956 gi | Serine (Or cysteine) proteinase inhibitor, clade B (Ovalbumin), memberDE 13,HU |
| Smp_006510 | 5902072 gi | Serine (Or cysteine) proteinase inhibitor, clade B (Ovalbumin), memberDE 3,Ser |
| SMP_119130 | 5902072 gi | Serine (Or cysteine) proteinase inhibitor, clade B (Ovalbumin), memberDE 3,squ |
| Smp_006510 | 40254871 gi,1 | Serine protease |
| Smp_112090 | 47551347 gi | Serine protease 1-like protein 1 |
| SMP_006510 | 47551347 gi | Serine protease 1-like protein 1,AAQ88957_chr19:636719-646430_L143P,GLGL782 |
| SMP_006510 | 148806923 gi | serine protease 45 [Homo sapiens] |

# Supplemental Information 1: Cercarial Elastase Interactions



| | | |
|---|---|---|
| Smp_006520 | 148806900 gi | serine protease 48 precursor [Homo sapiens] |
| Smp_006520 | 33695155 gi | Serine protease hepsin (EC 3.4.21.-) (Transmembrane protease, serineDE 1) [Con |
| SMP_006510 | 33695155 gi | Serine protease hepsin (EC 3.4.21.-) (Transmembrane protease, serineDE 1) [Con |
| Smp_112090 | 4506141 gi | Serine protease HTRA1 precursor (EC 3.4.21.-) (L56) |
| SMP_119130 | 4506141 gi | Serine protease HTRA1 precursor (EC 3.4.21.-) (L56),Serine protease HTRA1 precur |
| SMP_006510 | 4506141 gi | Serine protease HTRA1 precursor (EC 3.4.21.-) (L56),Serine protease HTRA1 precur |
| Smp_112090 | 7019477 gi | Serine protease HTRA2, mitochondrial precursor (EC 3.4.21.-) (HighDE temperatu |
| SMP_006510 | 7019477 gi | Serine protease HTRA2, mitochondrial precursor (EC 3.4.21.-) (HighDE temperatu |
| SMP_006510 | 94536774 gi | Serine protease inhibitor Kazal type 9 |
| SMP_006510 | 92110017 gi | serine protease inhibitor Kazal-type 13 precursor [Homo sapiens] |
| SMP_112090 | 7657453 gi | Serine protease inhibitor Kazal-type 4 precursor (Peptide PEC-60DE homolog),Se |
| SMP_006510 | 7657453 gi | Serine protease inhibitor Kazal-type 4 precursor (Peptide PEC-60DE homolog),Se |
| SMP_006510 | 74027261 gi | serine protease inhibitor Kazal-type 5 isoform b precursor [Homo sapiens] |
| SMP_006510 | 122937482 gi | serine protease inhibitor Kazal-type 8 precursor [Homo sapiens] |
| Smp_119130 | 110626171 gi | Serine protease inhibitor Kunitz type 3 |
| SMP_006510 | 110626171 gi | Serine protease inhibitor Kunitz type 3,Serine protease inhibitor Kunitz type 3 |
| SMP_112090 | 10863909 gi | Serine protease inhibitor, Kunitz type, 2,O43291_chr19:43447373-43474483_Q3H_C47 |
| SMP_006510 | 10863909 gi | Serine protease inhibitor, Kunitz type, 2,O43291_chr19:43447373-43474483_Q3H_C47 |
| SMP_006510 | 14602453 gi | serine protease,(P57727) Splice isoform B of P57727 |
| SMP_006510 | 40254871 gi | Serine protease,Transmembrane protease, serine 11E precursor (EC 3.4.21.-) (Seri |
| SMP_006510 | 122937297 gi | Serpin A11 precursor |
| SMP_006510 | 156071456 gi | serpin B11 [Homo sapiens] |
| SMP_112090 | 28076869 gi | Serpin B4 (Squamous cell carcinoma antigen 2) (SCCA-2) (Leupin),SQUAMOUS CELL CA |
| SMP_006510 | 28076869 gi | Serpin B4 (Squamous cell carcinoma antigen 2) (SCCA-2) (Leupin),SQUAMOUS CELL CA |
| SMP_119130 | 5453886 gi | Serpin I2 precursor (Myoepithelium-derived serine protease inhibitor)DE (Pancp |
| Smp_006510 | 5453886 gi | Serpin I2 precursor (Myoepithelium-derived serine protease inhibitor)DE (Pancp |
| SMP_006510 | 110225347 gi | SERPINA9 protein,Serpin peptidase inhibitor, clade A (Alpha-1 antiproteinase,DE |
| Smp_112090 | 31377629 gi | Similar to carboxypeptidase A5 |
| Smp_006520 | 24111255 gi | Similar to carboxypeptidase A6 |
| Smp_006510 | 14702169 gi | Similar to plasminogen activator, tissue,Similar to plasminogen activator, tissu |
| SMP_006510 | 39725934 gi | Similar to serine (or cysteine) proteinase inhibitor, clade F (alpha-2DE antip |
| SMP_006520 | 9790233 gi | Solute carrier organic anion transporter family member 1B3 (SoluteDE carrier f |
| SMP_006510 | 9790233 gi | Solute carrier organic anion transporter family member 1B3 (SoluteDE carrier f |
| SMP_006510 | 8394291 gi | Solute carrier organic anion transporter family member 1C1 (SoluteDE carrier f |
| Smp_006520 | 5032095 gi | Solute carrier organic anion transporter family member 2A1 (Solutecarrier family |
| SMP_006510 | 5032095 gi | Solute carrier organic anion transporter family member 2A1 (Solutecarrier family |

| | | |
|---|---|---|
| Smp_006520 | 13569932 gi | Solute carrier organic anion transporter family member 5A1 (Solutecarrier family |
| SMP_006510 | 13569932 gi | Solute carrier organic anion transporter family member 5A1 (Solutecarrier family |
| SMP_006510 | 93277099 gi | solute carrier organic anion transporter family member 6A1 [Homo sapiens] |
| SMP_119130 | 11545873 gi | SPARC-related modular calcium-binding protein 1 precursor (SecretedDE modular |
| SMP_006510 | 11545873 gi | SPARC-related modular calcium-binding protein 1 precursor (SecretedDE modular |
| Smp_119130 | 10863911 gi | SPINK2 protein (Fragment) |
| SMP_006510 | 10863911 gi | SPINK2 protein (Fragment),,Serine protease inhibitor Kazal-type 2 precursor (Acr |
| Smp_006520 | 6005882 gi | SPUVE protein |
| Smp_006520 | 11415040 gi | Suppressor of tumorigenicity 14 (EC 3.4.21.-) (Serine protease 14)DE (Matripta |
| SMP_006510 | 11415040 gi | Suppressor of tumorigenicity 14 (EC 3.4.21.-) (Serine protease 14)DE (Matripta |
| Smp_119130 | 148886654 gi | sushi, von Willebrand factor type A, EGF and pentraxin domain-containing protein |
| SMP_006510 | 113431326 gi | temporary entry |
| SMP_006510 | 55743098 gi,5! | temporary entry, |
| SMP_006510 | 118498341 gi | temporary entry, chymotrypsinogen B1 [Homo sapiens] |
| SMP_006510 | 29244926 gi | temporary entry, corin [Homo sapiens], |
| SMP_006510 | 148806900 gi | temporary entry, epidermis-specific serine protease-like protein [Homo sapiens] |
| SMP_006510 | 148231605 gi | temporary entry, ovochymase 2 [Homo sapiens] |
| SMP_006510 | 116256363 gi | temporary entry, transmembrane protease, serine 13 [Homo sapiens],transmembrane |
| SMP_006510 | 118498350 gi | temporary entry,cDNA FLJ77335, highly similar to Homo sapiens chymotrypsinogen B |
| SMP_006510 | 71834855 gi | temporary entry,kallikrein 3, (prostate specific antigen), isoform CRA_i |
| SMP_006510 | 110815798 gi | temporary entry,Ovochymase 1 |
| Smp_119130 | 4759164 gi | Testican-1 precursor (SPOCK protein) |
| SMP_006510 | 4759164 gi | Testican-1 precursor (SPOCK protein), |
| SMP_006510 | 93141003 gi | Testican-3 precursor (SPARC/osteonectin, CWCV, and Kazal-like domainsDE proteo |
| Smp_006510 | 33186882 gi | Testis serine protease 2 precursor,Testis serine protease 2 precursor,Testis ser |
| Smp_112090 | 7019563 gi | Testis-specific protease-like protein 50 precursor |
| SMP_006510 | 7019563 gi | Testis-specific protease-like protein 50 precursor, |
| Smp_006510 | 21614531 gi | testisin,testisin,(Q9Y6M0) Splice isoform 2 of Q9Y6M0 |
| Smp_112090 | 5803197 gi | Testisin precursor (EC 3.4.21.-) (Eosinophil serine protease 1) (ESP-DE 1) |
| SMP_006510 | 5803197 gi | Testisin precursor (EC 3.4.21.-) (Eosinophil serine protease 1) (ESP-DE 1),Tes |
| SMP_006510 | 4507377 gi | thyroxine-binding globulin precursor |
| SMP_119130 | 5730091 gi | Tissue factor pathway inhibitor 2,P48307_chr7:93128164-93132019_V102A_R231Q TISS |
| SMP_006510 | 5730091 gi | Tissue factor pathway inhibitor 2,P48307_chr7:93128164-93132019_V102A_R231Q TISS |
| SMP_006510 | 73760409 gi | Tissue factor pathway inhibitor beta, |
| SMP_119130 | 73760409 gi | Tissue factor pathway inhibitor beta,,Tissue factor pathway inhibitor beta |
| Smp_006510 | 4505861 gi | Tissue-type plasminogen activator precursor (EC 3.4.21.68) (tPA) (t-DE PA) (t- |

# Supplemental Information 1: Cercarial Elastase Interactions



| | | |
|---|---|---|
| Smp_112090 | 12383051 gi | TMEFF2 protein precursor (Transmembrane protein TENB2) (TPEF)DE (Transmembrane |
| SMP_006510 | 12383051 gi | TMEFF2 protein precursor (Transmembrane protein TENB2) (TPEF)DE (Transmembrane |
| Smp_119130 | 145701030 gi | TMPRSS3 |
| SMP_006510 | 145701030 gi | TMPRSS3,AAQ88894_chr11:117485670-117526277_R172Q_K193E_G203V |
| Smp_006510 | 169209600 gi | TPSAB1 protein,TPSAB1 protein,TPSAB1 protein.,TPS1 protein |
| Smp_119130 | 32698841 gi | Transmembrane protease, serine 11B (EC 3.4.21.-) |
| SMP_006510 | 32698841 gi | Transmembrane protease, serine 11B (EC 3.4.21.-),Putative uncharacterized protei |
| Smp_119130 | 4758508 gi | Transmembrane protease, serine 11D precursor (EC 3.4.21.-) (AirwayDE trypsin-l |
| SMP_006510 | 4758508 gi | Transmembrane protease, serine 11D precursor (EC 3.4.21.-) (AirwayDE trypsin-l |
| Smp_112090 | 32698940 gi | Transmembrane protease, serine 12 (EC 3.4.21.-) |
| SMP_006510 | 32698940 gi | Transmembrane protease, serine 12 (EC 3.4.21.-),Q86WS5_chr12:49523015-49567560_Y |
| Smp_006520 | 14602459 gi | Transmembrane protease, serine 2 precursor (EC 3.4.21.-) [Contains:DE Transmem |
| SMP_006510 | 14602459 gi | Transmembrane protease, serine 2 precursor (EC 3.4.21.-) [Contains:DE Transmem |
| Smp_112090 | 13173471 gi | Transmembrane protease, serine 3 (EC 3.4.21.-) (Serine protease TADG-DE 12) (T |
| SMP_006510 | 13173471 gi | Transmembrane protease, serine 3 (EC 3.4.21.-) (Serine protease TADG-DE 12) (T |
| Smp_006510 | 15451940 gi | Transmembrane protease, serine 4 (EC 3.4.21.-) (Membrane-type serineprotease 2) |
| Smp_006520 | 13540535 gi | Transmembrane protease, serine 5 (EC 3.4.21.-) (Spinesin) |
| SMP_006510 | 13540535 gi | Transmembrane protease, serine 5 (EC 3.4.21.-) (Spinesin),Transmembrane protease |
| SMP_006510 | 29568105 gi | Transmembrane protein with EGF-like and two follistatin-like domainsDE 1, |
| SMP_006520 | 29568105 gi | Transmembrane protein with EGF-like and two follistatin-like domainsDE 1,,Tran |
| SMP_006510 | 94400921 gi,9 | Trypsin domain containing 1 |
| SMP_006510 | 94400923 gi | Trypsin domain containing 1, |
| Smp_006510 | 48255915 gi | Trypsin X3 (KFIL2540),Trypsin X3 (KFIL2540), |
| Smp_112090 | 4506145 gi | Trypsinogen A |
| SMP_006510 | 4506145 gi | Trypsinogen A,Trypsinogen A (Protease, serine, 1) (Trypsin 1),Trypsin-1 precurso |
| Smp_112090 | 4506147 gi | Trypsinogen E (Protease, serine, 2) (Trypsin 2) (Anionic trypsinogen) |
| SMP_006510 | 4506147 gi | Trypsinogen E (Protease, serine, 2) (Trypsin 2) (Anionic trypsinogen),Trypsinoge |
| Smp_112090 | 13775595 gi | Tryptase beta-1 precursor (EC 3.4.21.59) (Tryptase-1) (Tryptase I) |
| SMP_006510 | 13775595 gi | Tryptase beta-1 precursor (EC 3.4.21.59) (Tryptase-1) (Tryptase I),Tryptase beta |
| Smp_112090 | 110578663 gi | tryptophan/serine protease [Homo sapiens] |
| SMP_006510 | 110578663 gi | tryptophan/serine protease [Homo sapiens], hypothetical protein LOC203074 [Homo |
| SMP_006510 | 110431331 gi | Type II transmembrane serine protease 7 precursor (HypotheticalDE protein FLJ1 |
| Smp_006510 | 6005820 gi | unnamed protein product,unnamed protein product,Solute carrier organic anion tra |
| Smp_112090 | 4505863 gi | Urokinase-type plasminogen activator (Plasminogen activator,DE urokinase) |
| SMP_006510 | 4505863 gi | Urokinase-type plasminogen activator (Plasminogen activator,DE urokinase),Plas |
| Smp_119130 | 5032223 gi | VESPR |

# Supplemental Information 1: Cercarial Elastase Interactions



| | | |
|---|---|---|
| Smp_006520 | 39930511 gi | VPPP1921 |
| SMP_006510 | 39930511 gi | VPPP1921,AAQ88713_chr18:55252112-55513543_V193G |
| Smp_006520 | 21703706 gi | WAP four-disulfide core domain 10A |
| SMP_006510 | 21703706 gi | WAP four-disulfide core domain 10A,,WAP four-disulfide core domain protein 10A p |
| Smp_119130 | 20069858 gi | WAP four-disulfide core domain 12 |
| SMP_006510 | 20069858 gi | WAP four-disulfide core domain 12,Q8WWY7_chr20:44437919-44438517_G23R,,WAP four- |
| Smp_006510 | 21717822 gi | WAP four-disulfide core domain 5,WAP four-disulfide core domain 5,probable prote |
| Smp_119130 | 153946389 gi | WAP four-disulfide core domain 8 |
| SMP_006510 | 153946389 gi | WAP four-disulfide core domain 8,Q8IUA0_chr20:44866097-44893315_N137 WAP four-di |
| Smp_119130 | 56699495 gi | WAP four-disulfide core domain protein 2 precursor (Major epididymis-DE specif |

# Supplemental Information 2: Venom Allergen Protein Interactions

| VAL Venom Allergen Proteins Interactions | | |
|---|---|---|
| S. mansoni | Human | Human Protein |
| Smp_123550 | 25121984 gi | BA719J20.1.2 (Acidic epididymal glycoprotein-like 1n),(P54107) Splice isoform Sh |
| Smp_123550 | 5174675 gi | Cysteine-rich secretory protein 3 precursor (CRISP-3) (SGP28 protein),Cysteine-r |
| Smp_123550 | 110825980 gi | Glioma pathogenesis-related protein,Glioma pathogenesis-related protein 1 precur |
| Smp_123550 | 70780384 gi | HGSC289 (OTTHUMP00000016309),Peptidase inhibitor 16,,HGSC289 |
| Smp_123550 | 30425410 gi | OTTHUMP00000031047 (R3H domain (Binds single-stranded nucleic acids)DE contain |
| Smp_123550 | 13899303 gi | Putative secretory protein (CocoaCrisp) (Trypsin inhibitor Hl)DE (Hypothetical |
| Smp_123550 | 4507671 gi | Testis-specific protein TPX1 e isoform (Testis-specific protein TPX1 bDE isofo |

# Supplemental Information 3: Calpain Interactions



## *Calpain Interactions*

| S. mansoni | Human | Human Protein |
|---|---|---|
| Smp_157500 | 27765074 gi | (P20807) Splice isoform II of P20807 |
| SMP_137410 | 27765074 gi | (P20807) Splice isoform II of P20807,AF127765_1 calpain 3; calcium activated neu |
| Smp_137410 | 27765076 gi | (P20807) Splice isoform IV of P20807 |
| Smp_137410 | 27765072 gi | AF127764_1 calpain 3; calcium activated neutral protease; CAPN3; CL1 |
| Smp_157500 | 13186302 gi | Calpain 10 (EC 3.4.22.17) (Calcium-activated neutral proteinase 10)DE (CANP 10 |
| Smp_157500 | 27765078 gi | Calpain 3, isoform e (CAPN3 protein) |
| Smp_157500 | 4502565 gi | Calpain small subunit 1 (CSS1) (Calcium-dependent protease smallDE subunit 1) |
| Smp_157500 | 41152101 gi | Calpain-13 (EC 3.4.22.-) |
| Smp_157500 | 4557405 gi | Calpain-3 (EC 3.4.22.54) (Calpain L3) (Calpain p94) (Calcium-activatedneutral pr |
| Smp_157500 | 13186316 gi | Calpain-6 (Calpamodulin) (CalpM) (Calpain-like protease X-linked) |
| SMP_137410 | 13186316 gi | Calpain-6 (Calpamodulin) (CalpM) (Calpain-like protease X-linked),Q9Y6Q1_chrX:10 |
| Smp_157500 | 7656959 gi | Calpain-7 (EC 3.4.22.-) (PalB homolog) (PalBH) |
| SMP_157500 | 5729758 gi | Calpain-9 (EC 3.4.22.-) (Digestive tract-specific calpain) (nCL-4)DE (CG36 pro |
| Smp_137410 | 12408656 gi | Cell proliferation-inducing protein 30 |
| Smp_157500 | 20149675 gi | EF hand domain containing 2 |
| Smp_157500 | 21361462 gi | EH-domain containing 2 (EH domain-containing protein-2) |
| Smp_137410 | 30240932 gi | EH-domain-containing protein 1 (Testilin) (hPAST1) |
| Smp_157500 | 4503593 gi | Epidermal growth factor receptor pathway substrate 15 |
| Smp_157500 | 10864047 gi | Epidermal growth factor receptor substrate EPS15R |
| Smp_137410 | 7705383 gi | GC36 protein |
| SMP_089460.: | 7705383 gi | GC36 protein, |
| Smp_157500 | 157389005 gi | Hypothetical protein |
| Smp_137410 | 4507207 gi | Hypothetical protein DKFZp459G0314 |
| Smp_157500 | 38707981 gi | Hypothetical protein FLJ45489 |
| SMP_089460.: | 38707981 gi | Hypothetical protein FLJ45489, |
| Smp_137410 | 6912388 gi | Hypothetical protein GCA |
| Smp_137410 | 48476342 gi | Mitochondrial ATP-Mg/Pi carrier (Mitochondrial Ca2+-dependent soluteDE carrier |
| Smp_157500 | 113865939 gi | Novel S100 calcium-binding protein (S100 calcium binding protein A7-DE like 2) |
| Smp_157500 | 6912582 gi | Peflin (PEF protein) (Penta-EF hand domain containing 1) (CDNADE FLJ10558 fis, |
| Smp_137410 | 113411704 gi | PREDICTED: calpain 8 [Homo sapiens] |
| Smp_137410 | 88943801 gi | PREDICTED: similar to calpain 8 [Homo sapiens] |
| Smp_157500 | 88952798 gi | PREDICTED: similar to calpain isoform 4 [Homo sapiens] |

# Supplemental Information 3: Calpain Interactions

| | | |
|---|---|---|
| Smp_137410 | 7019485 gi | Programmed cell death 6 |
| Smp_157500 | 28827815 gi | S100 calcium binding protein A15 (S100 calcium binding protein A7-likeDE 1) |
| Smp_137410 | 14161692 gi | Similar to RIKEN cDNA 2310005G05 gene |
| Smp_157500 | 5032105 gi | Small optic lobes homolog |
| SMP_089460.: | 5032105 gi | Small optic lobes homolog,O75808_chr16:536840-543514_G19C SMALL OPTIC LOBES HOMO |
| Smp_137410 | 38679884 gi | sorcin isoform B [Homo sapiens] |
| Smp_157500 | 47078247 gi | temporary entry |
| SMP_089460.: | 47078247 gi | temporary entry, |
| Smp_137410 | 31377581 gi | Uncharacterized protein C17orf57 |
| Smp_137410 | 37577157 gi | Unknown (protein for MGC:9155 |
| SMP_089460.: | 37577157 gi | Unknown (protein for MGC:9155,Calpain-5 (EC 3.4.22.-) (nCL-3) (htra-3),,Calpain |
| Smp_157500 | 89353291 gi,5 | unnamed protein product |

# Supplemental Information 4: Cystatin Interactions



| Cystatin Interactions | | |
|---|---|---|
| | | |
| S. mansoni | Human | Human Protein |
| SMP_006390 | 4503139 gi | Cathepsin B, preproprotein,Cathepsin B precursor (EC 3.4.22.1) (Cathepsin B1) (A |
| SMP_006390 | 6042196 gi | Cathepsin F precursor (EC 3.4.22.41) (CATSF),Q9UBX1_chr11:66106767-66111317_Q153 |
| SMP_006390 | 4503151 gi | Cathepsin K precursor (EC 3.4.22.38) (Cathepsin O) (Cathepsin X)DE (Cathepsin |
| SMP_006390 | 23110960 gi | Cathepsin L2,CATHEPSIN L2 PRECURSOR (EC 3.4.22.43) (CATHEPSIN V) (CATHEPSIN U),, |
| SMP_006390 | 4557501 gi | Cathepsin O precursor (EC 3.4.22.42),Cathepsin O precursor (EC 3.4.22.42)., |
| SMP_006390 | 23110962 gi | Cathepsin S,P25774_chr1:147922107-147953821_S161 CATHEPSIN S PRECURSOR (EC 3.4.2 |
| SMP_006390 | 22538442 gi | Cathepsin Z precursor (EC 3.4.22.-) (Cathepsin X) (Cathepsin P), |
| SMP_006390 | 4503155 gi | CTSL protein (Cathepsin L) (Hypothetical protein DKFZp686A18159),CTSL protein (H |
| SMP_006390 | 23110955 gi | pro-cathepsin H preproprotein [Homo sapiens] |

# Supplemental Information 5: Tetraspanin Interactions

| Tetraspanin Interactions | | |
|---|---|---|
| S. mansoni | Human | Human Protein |
| Smp_100080 | 5032207 gi | (Q96QS1) Splice isoform 2 of Q96QS1, tetraspanin |
| Smp_181530 | 4757944 gi | CD81 antigen (26 kDa cell surface protein TAPA-1) (Target of theDE antiprolife |
| Smp_100080 | 21264580 gi | Hypothetical 24.1 kDa protein, tetraspanin |
| Smp_100080 | 145580605 gi | tetraspanin |
| Smp_181530 | 145580605 gi | tetraspanin |

# Supplemental Information 6: Human Immunoglobin Interactions

| Human Immunoglobin Interactions | | | |
|---|---|---|---|
| S. mansoni | S. mansoni Protein | Human | Human Protein |
| SMP_027400 | Ankyrin, kinase,Serine/ | 13994242 gi | C-Cbl-interacting protein ige |
| SMP_031880 | Immunoglobulin-like | 21361212 gi | CTLA4,CYTOTOXIC T-LYMPHOCYTE PROTEIN 4 |
| SMP_032970 | Calcium-binding EF-han | 156616292 gi | OTTHUMP00000016605 ige |
| SMP_043990.: | basigin | 146229333 gi | allergin-1 precursor [Homo sapiens] |
| SMP_055500 | DNA-directed DNA polyr | 21361212 gi | CTLA4 IgG Proteins |
| SMP_082490 | Cyclin, A/B/D/E,Cyclin, | 112382241 gi | Proto-oncogene tyrosine-protein kinase |
| SMP_115130 | Immunoglobulin-like | 23312371 gi | CD40 type II isoform, |
| SMP_127420 | Cystathionine beta-synt | 156104869 gi | Chloride channel protein ige |
| SMP_129430 | Transcription factor, MA | 19923215 gi | Myocyte-specific enhancer factor 2C IgG Proteins |
| SMP_133590 | C2 calcium-dependent n | 60097902 gi | Filaggrin ige |
| SMP_141410 | Carbohydrate kinase, FC | 11231177 gi | AF109683_1 leukocyte-associated Ig-like receptor 1b ige |
| SMP_148450 | Chitinase II,Glycoside h | 144226251 gi | (cartilage glycoprotein-39) ige |
| SMP_153170 | Fibronectin, type III | 63055063 gi | Fc gamma receptor I IgG Proteins |
| SMP_156630 | Protein coding gene | 50511926 gi | FCGR2B protein (Fragment) IgG Proteins |
| SMP_169240 | SH2 motif,Src,Variant SI | 21618338 gi | (P40763) Splice isoform Del-701 of P40763 ige |
| SMP_172070 | Protein coding gene | 23312371 gi | CD40 type II isoform IgG Proteins |
